# Effects of evolocumab in individuals with type 2 diabetes with and without atherogenic dyslipidemia: An analysis from BANTING and BERSON

**DOI:** 10.1186/s12933-021-01287-6

**Published:** 2021-04-30

**Authors:** Alberto J. Lorenzatti, Maria Laura Monsalvo, J. Antonio G. López, Huei Wang, Robert S. Rosenson

**Affiliations:** 1 Clinical Research and Cardiology, Instituto Médico DAMIC/Fundación Rusculleda, Córdoba, Argentina; 2 Amgen Inc, Thousand Oaks, CA USA; 3 Amgen Inc, Newbury Park, CA USA; 4 Icahn School of Medicine At Mount Sinai, New York, NY USA

**Keywords:** Atherogenic dyslipidemia, Cardiovascular disease, Diabetes mellitus, Evolocumab, Lipid-lowering therapy, Lipoproteins, PCSK9 inhibition, Treatment goals

## Abstract

**Background:**

Atherogenic dyslipidemia (AD), characterized by increased concentrations of apolipoprotein B (ApoB)-containing particles, is often present in individuals with type 2 diabetes mellitus (T2DM). Non-high-density lipoprotein cholesterol (non-HDL-C), cholesterol transported by apolipoprotein B (ApoB)-containing particles), and total apoB are considered secondary goals of lipid-lowering therapy to guide treatment of residual cardiovascular risk. The BANTING and BERSON studies demonstrated that evolocumab added to statin therapy reduced atherogenic lipid and lipoproteins concentrations in patients with T2DM.

**Methods:**

This post-hoc analysis combined data from two randomized, placebo-controlled trials, BANTING and BERSON, to investigate the effect of evolocumab (140 mg every two weeks [Q2W] or 420 mg monthly [QM]) on atherogenic lipid (LDL-C, non-HDL-C, VLDL-C, remnant cholesterol) and lipoproteins (ApoB, lipoprotein(a) (Lp[a])), and achievement of 2019 European Society of Cardiology/European Atherosclerosis Society lipid treatment goals in individuals with and without AD.

**Results:**

In individuals with high TGs with (n = 389) and without (n = 196) AD receiving background statin therapy, evolocumab, compared with placebo, substantially reduced the cholesterol levels from all ApoB atherogenic lipoproteins (least squares (LS) mean LDL-C by 66.7% to 74.3%, non-HDL-C by 53.4% to 65.8%, median remnant cholesterol by 28.9% to 34.2%, VLDL-C by 16.1% to 19.6%) and median TGs levels (by 17.5% to 19.6%) at the mean of weeks 10 and 12. LS mean ApoB was significantly reduced by 41.5% to 56.6% at week 12. Results were consistent in diabetic individuals with normal TGs (n = 519). Evolocumab was also associated with a significant reduction in median Lp(a) by 35.0% to 53.9% at the mean of weeks 10 and 12. A majority (74.7% to 79.8%) of evolocumab-treated individuals achieved the goal of both an LDL-C < 1.4 mmol/L and an LDL-C reduction of at least 50%, > 75% achieved non-HDL-C < 2.2 mmol/L at the mean of weeks 10 and 12, and > 67% achieved ApoB < 65 mg/dL at week 12.

**Conclusions:**

Evolocumab effectively reduced LDL-C, non-HDL-C, ApoB, Lp(a), and remnant cholesterol in individuals with T2DM with and without AD. Evolocumab Q2W or QM enabled most individuals at high/very-high cardiovascular disease risk to achieve their LDL-C, non-HDL-C, and ApoB recommended goals.

**Supplementary Information:**

The online version contains supplementary material available at 10.1186/s12933-021-01287-6.

## Background

The lipid profile of patients with atherogenic dyslipidemia (AD) is generally characterized by high triglycerides (TGs), low high-density lipoprotein cholesterol (HDL-C), and mildly elevated or even normal low-density lipoprotein cholesterol (LDL-C) levels. The lipoprotein assessment in these patients reveals an increase in triglyceride-rich lipoproteins (TRL) (chylomicrons, very-low-density lipoprotein [VLDL]), accumulation of lipoprotein remnants (chylomicron remnants, small VLDL, and intermediate-density lipoprotein), and predominantly small, dense LDL particles [1, 2]. The atherogenic nature is due to the increased number of apolipoprotein B (ApoB)-containing particles, also characterized by elevated non-HDL-C levels. Insulin resistance has been identified as the main factor for the development of AD, which is often present in patients with insulin resistance phenotypically captured by obesity, metabolic syndrome, impaired glucose tolerance, and type 2 diabetes mellitus (T2DM) [3–5].

Epidemiological, clinical, and genetic studies have supported the AD’s role as a causal factor for the development and progression of atherosclerotic cardiovascular disease (ASCVD) [6–12]. Many patients who attained their LDL-C goal with lipid-lowering therapies continue to experience cardiovascular events; the increase of other ApoB-containing lipoproteins likely contributes to the residual risk [13–18]. Thus, while LDL-C is the primary focus for dyslipidemia management and ASCVD prevention, it might not reflect the actual atherogenic burden in patients with AD. In these patients, measuring both non-HDL-C and ApoB levels is recommended for ASCVD risk assessment [5]. Moreover, the 2019 European Society of Cardiology/European Atherosclerosis Society (ESC/EAS) guidelines for the management of dyslipidemias have defined secondary goals for both non-HDL-C and ApoB to help guide lipid-lowering therapy adjustments after the achievement of an LDL-C goal [5].

Patients with type 2 diabetes mellitus (T2DM) are at exceptionally high risk of cardiovascular disease (CVD) morbidity and mortality; several society guidelines recommend an aggressive lipid management approach to reduce CVD risk [5, 19–21]. However, lipid levels in many patients with T2DM receiving statin therapy remain above the recommended thresholds. A second therapy is needed to attain lipid treatment goals and further reduce the risk of cardiovascular events [22, 23]. In two double-blind, randomized, phase 3 studies of individuals with T2DM (BANTING, NCT02739984; BERSON, NCT02662569), evolocumab, a fully human monoclonal antibody that is a proprotein convertase subtilisin/kexin type 9 inhibitor (PCSK9i), significantly reduced LDL-C and other atherogenic lipid parameters when added on to statin therapy of at least moderate intensity [24, 25]. In two prespecified analyses of the FOURIER trial (NCT01764633), evolocumab also reduced the risk of major cardiovascular events (myocardial infarction, stroke, and coronary revascularization) with similar efficacy in patients with or without diabetes [26] and in patients with metabolic syndrome with or without diabetes [27] who were treated with a maximally tolerated dose of statin. Patients with established ASCVD also had a lower risk of developing complex coronary artery disease requiring revascularization (complex PCI or CABG) when evolocumab was added to statin therapy [28]. The benefits, which increased over time, extended to patients with diabetes in whom the risk was significantly reduced by 36%.

In patients with T2DM at high or very high CVD risk who cannot attain their LDL-C goal with a maximally tolerated dose of statin in combination with ezetimibe, or in whom statins are not tolerated, the addition of a PCSK9i is recommended to achieve an LDL-C < 1.8 mmol/L (high-risk patients) or < 1.4 mmol/L (very high-risk patients) and reduce LDL-C by at least 50% [19]. The lipid-lowering effect of evolocumab in the subgroup of T2DM patients with AD (high TGs and/or low HDL-C) has not been previously reported. The objective of this post-hoc analysis was to investigate the effect of evolocumab on ApoB, on cholesterol levels carried by ApoB-containing lipoproteins, on HDL-C, and on the achievement of 2019 ESC/EAS [5] lipid and lipoprotein treatment goals in patients with T2DM with and without AD.

## Methods

This analysis included pooled data from two randomized, double-blind, 12-week studies of evolocumab vs placebo treatment, on background statin therapy of at least moderate intensity, in patients with T2DM and hypercholesterolemia or mixed dyslipidemia (BANTING [NCT02739984] [25] and BERSON [NCT02662569] [24]). For both studies, detailed methods, participant inclusion/exclusion criteria, and primary results have been previously published [24, 25, 29]. All procedures followed Good Clinical Practice, the protocols were approved by institutional review boards at participating sites, and all participants provided written informed consent at enrollment. An independent ethics committee or Ethics Review Board approved study protocols for all studies contributing data to this post-hoc analysis.

Participants in both studies were ≥ 18 years of age, diagnosed with T2DM, had hemoglobin A1c ≤ 10%, and had been on stable pharmacological therapy for diabetes for ≥ 6 months. Fasting TGs were limited to ≤ 4.5 mmol/L in BERSON and ≤ 6.8 mmol/L in BANTING. Before randomization in BANTING [25], individuals without known clinical CVD were required to have a fasting LDL-C ≥ 2.59 mmol/L or non-HDL-C ≥ 3.39 mmol/L Individuals with known clinical CVD (defined as a history of myocardial infarction, stable or unstable angina, coronary or other arterial revascularization, stroke, transient ischemic attack, or peripheral arterial disease presumed to be of atherosclerotic origin) were required to have a fasting LDL-C ≥ 1.81 mmol/L or non-HDL-C ≥ 2.59 mmol/L. In BERSON [24], individuals on statin therapy at screening were required to have an LDL-C ≥ 2.6 mmol/L. Individuals who were not on statin therapy at screening were required to have an LDL-C ≥ 3.4 mmol/L. Blood samples were obtained after fasting for a minimum of 9 h.

In both studies, all participants were required to be on background statin therapy. In BANTING [25], individuals receiving maximally tolerated doses of statins of at least moderate intensity were randomized to receive evolocumab 420 mg subcutaneous (SC) or placebo monthly (QM). In BERSON [24], participants who initiated atorvastatin 20 mg at enrollment and then were randomized to receive evolocumab 140 mg SC every 2 weeks (Q2W) or 420 mg SC QM or placebo Q2W or QM.

For the current analysis, individuals in the pooled full analysis sets of both studies (ie, participants who were randomized and received at least one dose of study drug [placebo or evolocumab]) were divided into three groups as follows:

(1) individuals with high TGs and normal HDL-C (i.e. without AD), (2) individuals with high TGs and low HDL-C (i.e. with AD), and (3) individuals with normal TGs and normal HDL-C levels. High TGs were defined as baseline TGs ≥ 1.69 mmol/L, and low HDL-C was defined for male individuals as baseline HDL-C < 1.03 mmol/L and female individuals as baseline HDL-C < 1.29 mmol/L [30].

The pooled analysis endpoints of interest were percentage change from baseline and change from baseline in lipid values (LDL-C, non-HDL-C, ApoB, lipoprotein(a) [Lp(a)], VLDL-C, remnant cholesterol, TGs, and HDL-C). In addition, we evaluated the percentage of individuals who attained lipid goals per the 2019 ESC/EAS guidelines for patients at high or very-high CVD risk [5]. The goals assessed were the following: ≥ 50% reduction in LDL-C, LDL-C < 1.4 mmol/L, ≥ 50% reduction in LDL-C and LDL-C < 1.4 mmol/L, LDL-C < 1.0 mmol/L, non-HDL-C < 2.6 mmol/L, non-HDL-C < 2.2 mmol/L, ApoB < 80 mg/dL, and ApoB < 65 mg/dL. The correlation of ApoB100 with non-HDL-C, remnant cholesterol and TGs in patients treated with evolocumab (Q2W and QM combined) was also assessed.

Lipid and lipoprotein endpoints were measured at week 10 and week 12. The outcomes reported here are the means of weeks 10 and 12 except for total ApoB, which was not measured at week 10 in BERSON, so the results are reported at week 12. ApoB100 was measured only in BERSON, per protocol, at weeks 10 and 12. The final study drug dose was at week 8 in the BANTING study; for the BERSON study, it was at week 8 for QM participants and week 10 for Q2W participants. The maximum reduction of LDL-C occurs approximately 2 weeks after the dose for individuals receiving evolocumab 420 mg QW [31]; therefore, the mean of weeks 10 and 12 incorporates time points that would likely represent maximum LDL-C reduction for some individuals, depending on their dose regimen, combined with a time point at which LDL-C levels are starting to return to baseline. The lipid endpoints at week 12 were also analyzed; the data are not reported here.

LDL-C was calculated using the Friedewald formula. When the calculated LDL-C was < 1.0 mmol/L or TGs were > 4.5 mmol/L, calculated LDL-C was replaced with ultracentrifugation to inform LDL-C and VLDL-C from the same blood sample, if available. In both studies, total ApoB was measured by immunoturbidimetry and ELISA measured ApoB48, both at Medpace (Cincinnati, Ohio; Leuven, Belgium). For the BERSON study, because the protocol required ApoB100 and ApoB48 results separately, for this analysis, total ApoB was the summation of ApoB100 and ApoB48 from the same blood sample. Remnant cholesterol was calculated as total cholesterol minus LDL-C and HDL-C from the same blood sample [9, 32].

### Statistical analysis

Analysis of each endpoint comparing evolocumab vs placebo was conducted within each of the three groups (high TGs and normal HDL-C, high TGs and low HDL-C, normal TGs and normal HDL-C) and two dosing regimens (Q2W and QM) or both combined if sample size was small. For analyses of change and percentage change from baseline for endpoints that followed normal distributed data, treatment group differences (evolocumab minus placebo) were evaluated with a repeated measures linear effects model which included study, treatment group, scheduled visit, and the interaction of treatment group with scheduled visit as covariates. For analyses of change and percentage change from baseline for endpoints with skewed and non-normal distributed data, the median difference and 95% CI are obtained from McKean-Schrader algorithm, and P-value is obtained from Quade test adjusting for baseline value. In addition, the relationship between changes in ApoB100 with changes in non-HDL-C, TGs, and remnant cholesterol within evolocumab was explored. The scatter plots, Pearson correlation coefficients, and associated *P*-values were generated for changes on the mean of weeks 10 and 12 from baseline in ApoB100 with changes on the mean of weeks 10 and 12 from baseline in non-HDL-C, TGs and remnant cholesterol for evolocumab for the overall and within each group. Spearman’s rank correlation was used if the data was non-normal distributed. For the goal attainment analysis, treatment group differences were evaluated with Cochran-Mantel–Haenszel tests, stratified by study. Missing values were considered as non-attainment. Alpha levels were not adjusted for multiplicity. All analyses were performed using SAS version 9.4 (SAS Institute, Cary, NC, USA).

## Results

The pooled BERSON and BANTING full analysis sets included 1402 participants (placebo, n = 465; evolocumab, n = 937). A total of 1104 participants met the TGs and HDL-C criteria as follows: 196 participants (17.8%) had high TGs and normal HDL-C; 389 participants (35.2%) had both high TGs and low HDL-C, and 519 participants (47.0%) had normal TGs and normal HDL-C.

### Baseline characteristics

Baseline lipid profile, disease, and demographic variables are shown in Table 1 (detailed results by dose regimen are shown in, Additional file 1: Table S1). Baseline characteristics were generally well balanced between individuals allocated to placebo or evolocumab within each group. In all three groups, fasting serum glucose and duration of diabetes were higher in individuals allocated to evolocumab than to placebo. As expected, some baseline characteristics of the groups differed in some respects. Body mass index, waist circumference, any statin use, and high-intensity statin use were higher in the two groups with high TGs than in the group with normal TGs and normal HDL-C. LDL-C was higher in the group with high TGs without AD. Non-HDL-C, ApoB, VLDL-C, and remnant cholesterol were higher in the two groups of individuals with high TGs than in the group with normal TGs and normal HDL-C. Lp(a) was generally higher in the group with normal TGs and normal HDL-C. The percentage of Black or African American individuals was also higher in this group. Notably, individuals in the two groups with high TGs were more likely to be White and report Hispanic/Latino ethnicity than were individuals with normal TGs.

**Table 1:** Patient demographic and disease characteristics at baseline

Characteristic	High TGs and normal HDL-C		High TGs and low HDL-C		Normal TGs and normal HDL-C	
	Placebo N = 64	Evolocumab N = 132	Placebo N = 125	Evolocumab N = 264	Placebo N = 178	Evolocumab N = 341
Sex, female, n (%)	28 (43.8)	58 (43.9)	72 (57.6)	143 (54.2)	95 (53.4)	161 (47.2)
Age, years, mean (SD)	63.0 (8.6)	61.9 (8.6)	60.2 (8.2)	60.9 (8.5)	62.6 (8.8)	62.2 (8.1)
Ethnicity Hispanic/Latino, n (%)	12 (18.8)	28 (21.2)	29 (23.2)	60 (22.7)	23 (12.9)	55 (16.1)
Race, n (%)						
White	40 (62.5)	82 (62.1)	79 (63.2)	165 (62.5)	83 (46.6)	157 (46.0)
Asian	15 (23.4)	40 (30.3)	32 (25.6)	73 (27.7)	72 (40.4)	144 (42.2)
Black or African American	7 (10.9)	8 (6.1)	6 (4.8)	11 (4.2)	20 (11.2)	31 (9.1)
American Indian or Alaska native	1 (1.6)	1 (0.8)	6 (4.8)	6 (2.3)	0 (0.0)	2 (0.6)
Native Hawaiian or other Pacific Islander	0 (0.0)	0 (0.0)	0 (0.0)	1 (0.4)	0 (0.0)	0 (0.0)
Multiple	1 (1.6)	1 (0.8)	2 (1.6)	7 (2.7)	3 (1.7)	6 (1.8)
Other	0 (0.0)	0 (0.0)	0 (0.0)	1 (0.4)	0 (0.0)	1 (0.3)
BMI, kg/m^2^, mean (SD)	31.2 (7.4)	30.2 (6.0)	31.2 (6.6)	31.6 (6.3)	28.8 (6.2)	28.4 (5.2)
Waist circumference, cm, mean (SD)	103.9 (17.2)	103.2 (14.4)	104.1 (16.6)	104.2 (15.7)	96.8 (14.4)	97.3 (12.8)
Duration of diabetes, years, mean (SD)	9.6 (7.51)	10.6 (7.80)	9.9 (7.52)	10.5 (7.18)	10.1 (7.75)	10.2 (7.60)
Fasting serum glucose, mmol/L, median (Q1, Q3)	7.25 (6.00, 8.85)	7.30 (6.20, 9.50)	7.50 (6.20, 9.70)	8.15 (6.35, 10.50)	6.80 (5.80, 8.30)	7.00 (5.80, 8.70)
HbA1c (fraction of 1), median (Q1, Q3)	0.0690 (0.0640, 0.0755)	0.0700 (0.0645, 0.0815)	0.0740 (0.0640, 0.0860)	0.0740 (0.0655, 0.0840)	0.0670 (0.0620, 0.0760)	0.0700 (0.0630, 0.0800)
Cardiovascular risk category per 2019 ESC/EAS [5] guidelines, n (%)						
Very high risk	58 (90.6)	124 (93.9)	116 (92.8)	243 (92.0)	154 (86.5)	303 (88.9)
High risk	6 (9.4)	8 (6.1)	9 (7.2)	21 (8.0)	24 (13.5)	38 (11.1)
Statin use per 2018 ACC/AHA guidelines [21], n (%)						
Any statin use^a^	48 (75.0)	104 (78.8)	95 (76.0)	213 (80.7)	120 (67.4)	216 (63.3)
High-intensity statin	12 (18.8)	29 (22.0)	35 (28.0)	67 (25.4)	23 (12.9)	45 (13.2)
Moderate-intensity statin	35 (54.7)	71 (53.8)	56 (44.8)	134 (50.8)	93 (52.2)	162 (47.5)
Ezetimibe	1 (1.6)	4 (3.0)	4 (3.2)	7 (2.7)	5 (2.8)	9 (2.6)
Lipid concentration						
LDL-C, mmol/L, mean (SD)	2.811 (0.901)	2.737 (0.971)	2.508 (0.817)	2.599 (0.895)	2.539 (0.884)	2.511 (0.818)
Non-HDL-C, mmol/L, mean (SD)	3.819 (0.977)	3.752 (1.004)	3.720 (0.933)	3.793 (0.985)	3.052 (0.926)	3.033 (0.849)
Total ApoB, g/L, mean (SD)	0.986 (0.225)	0.974 (0.229)	0.983 (0.238)	0.996 (0.239)	0.825 (0.217)	0.827 (0.205)
Lp(a), nmol/L, median (Q1, Q3)	21.0 7.0, 58.0	37.0 9.0, 128.8	29.0 8.0, 136.0	22.0 8.0, 76.0	47.0 12.0, 128.0	33.0 11.5, 121.0
VLDL-C, mmol/L, median (Q1, Q3)	0.970 (0.830, 1.140)	0.953 (0.840, 1.110)	1.040 (0.880, 1.335)	1.090 (0.910, 1.370)	0.520 (0.410, 0.600)	0.520 (0.410, 0.645)
Remnant cholesterol, mmol/L, median (Q1, Q3)	0.965 (0.830, 1.125)	0.953 (0.840, 1.120)	1.060 (0.875, 1.400)	1.100 (0.900, 1.370)	0.520 (0.420, 0.600)	0.520 (0.420, 0.640)
TG, mmol/L, median (Q1, Q3)	2.133 1.815, 2.475	2.078 1.830, 2.445	2.300 1.940, 3.065	2.440 1.975, 3.038	1.115 0.915, 1.300	1.140 0.900, 1.390
HDL-C, mmol/L, mean (SD)	1.353 (0.245)	1.343 (0.206)	0.947 (0.163)	0.935 (0.185)	1.488 (0.338)	1.440 (0.286)

When the calculated LDL-C was < 1.0 mmol/L or TGs were > 4.5 mmol/L, calculated LDL-C was replaced with ultracentrifugation to inform LDL-C and VLDL-C from the same blood sample, if available

*ACC* American College of Cardiology, *AHA* American Heart Association, *ApoB* apolipoprotein B, *BMI* body mass index, *EAS* European Atherosclerosis Society, *ESC* European Society of Cardiology, *HbA1c* hemoglobin A1c, *HDL-C* high-density lipoprotein cholesterol, *LDL-C* low-density lipoprotein cholesterol, *Lp(a)* lipoprotein(a), *non-HDL-C* non-high-density lipoprotein cholesterol, *Q* quartile, *TG* triglycerides, *VLDL-C* very low-density lipoprotein cholesterol

^a^Represents use of statin therapy before initiating any treatment intervention (including atorvastatin and/or study drug); in BANTING, 99.3% of patients were on moderate- or high-intensity statin at randomization; in BERSON, all patients initiated moderate-intensity atorvastatin at enrollment

### Lipid outcomes

#### Percentage change from baseline

**Fig. 1:**
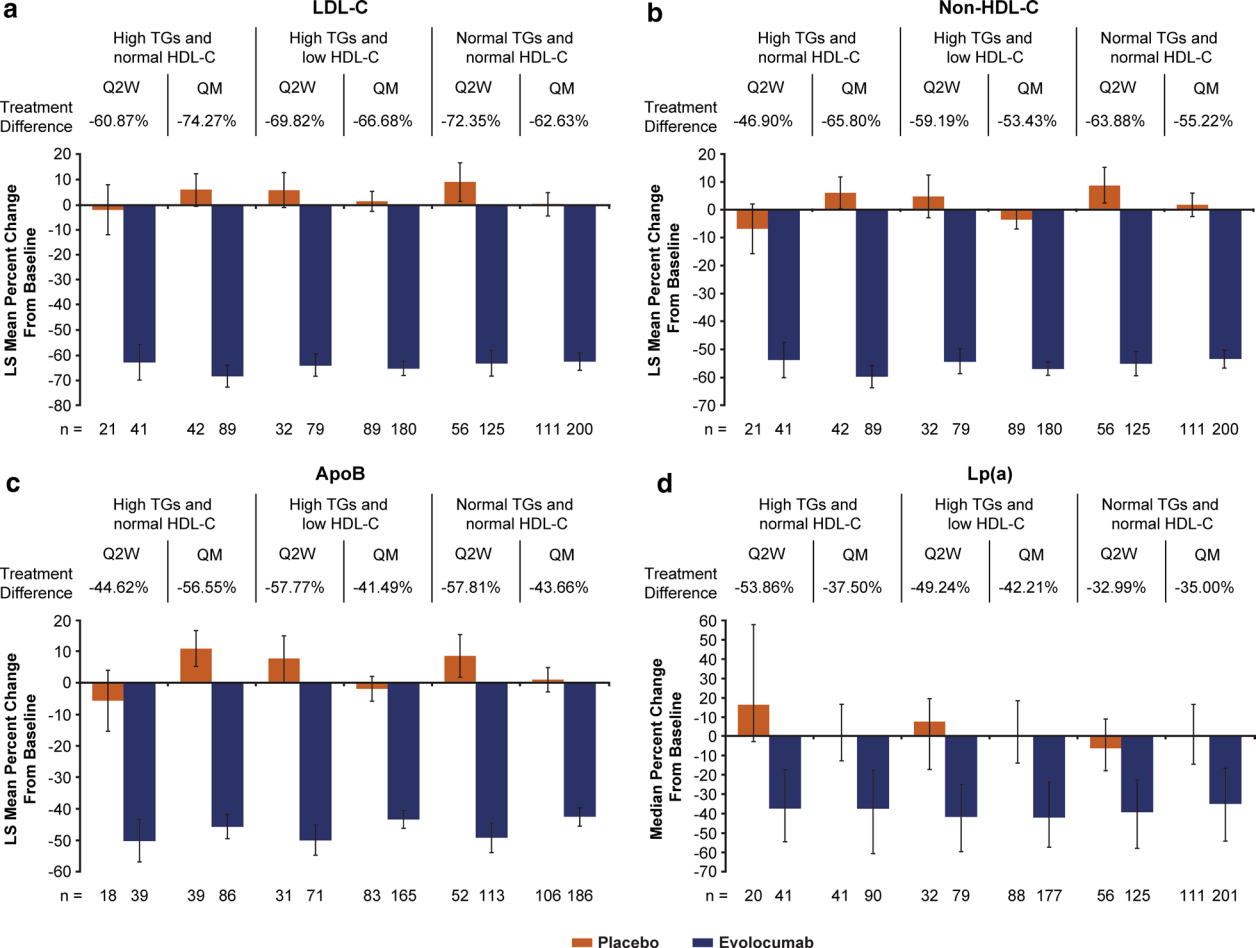
Percentage change from baseline in ApoB and ApoB-lipoprotein cholesterol with evolocumab vs placebo on background statin therapy. Values shown are means of weeks 10 and 12 except for ApoB, which was measured at week 12. All treatment differences between evolocumab and placebo are statistically significant at *P* < 0.0001. When the calculated LDL-C was < 1.0 mmol/L or TGs were > 4.5 mmol/L, calculated LDL-C was replaced with ultracentrifugation to inform LDL-C and VLDL-C from the same blood sample, if available. *ApoB* apolipoprotein B, *HDL-C* high-density lipoprotein cholesterol, *LDL-C* low-density lipoprotein cholesterol, *Lp(a)* lipoprotein(a), *non-HDL-C* non-high-density lipoprotein cholesterol, *Q2W* every 2 weeks, *QM* monthly, *TG* triglycerides, *VLDL-C* very low-density lipoprotein cholesterol

**Fig. 2:**
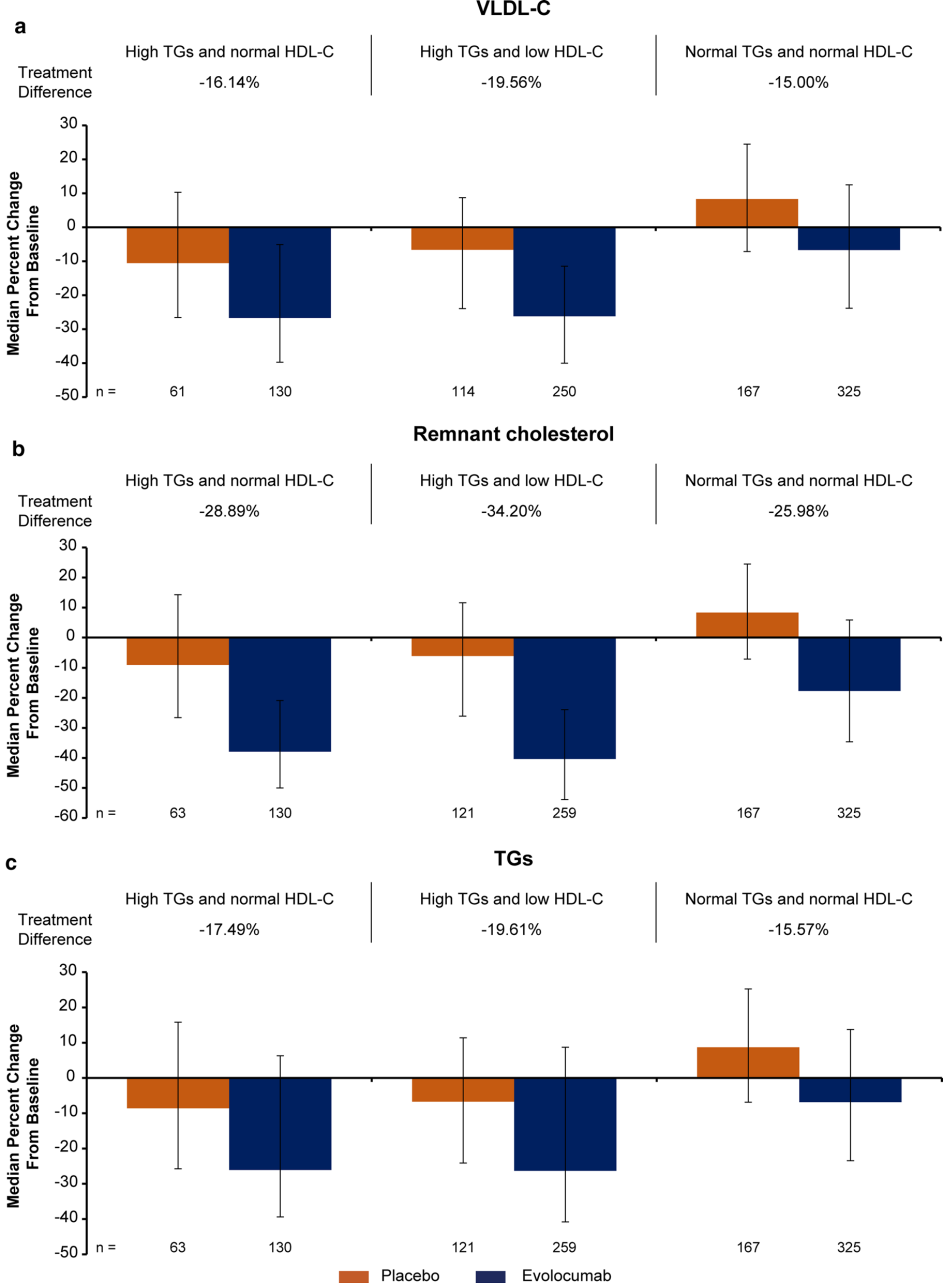
Percentage change from baseline in VLDL-C, remnant cholesterol and TGs with evolocumab vs placebo in participants with high TGs with or without AD on background statin therapy. All treatment differences between evolocumab and placebo are statistically significant at *P* < 0.0001 except for VLDL-C in the group with high TGs and normal HDL-C (*P* = 0.0002). *HDL-C* high-density lipoprotein cholesterol, *TG* triglycerides, *VLDL-C* very low-density lipoprotein cholesterol

On top of background statin therapy, individuals treated with evolocumab, compared with placebo, had greater percentage change from baseline across all lipid parameters, with consistent results across dosing regimens and in individuals with and without high TGs (Figs. 1, 2 and Additional file 1: Table S2). At the mean of weeks 10 and 12, compared with placebo, evolocumab treatment reduced LDL-C by a least squares (LS) mean of 60.9% (95% confidence interval [CI], 48.6, 73.2) with Q2W dosing and 74.3% (95% CI 66.6, 81.9) with QM dosing in the group with high TGs and normal HDL-C, 69.8% (95% CI 61.6, 78.1) with Q2W dosing and 66.7% (95% CI 61.9, 71.5) with QM dosing in the group with high TGs and low HDL-C, and 72.4% (95% CI 63.2, 81.5) with Q2W dosing and 62.6% (95% CI 57.0, 68.3) with QM dosing in the group with normal TGs and normal HDL-C, all *P-*values < 0.0001. Compared with placebo, evolocumab treatment reduced non-HDL-C by an LS mean of 46.9% (95% CI 36.0, 57.9) with Q2W dosing and 65.8% (95% CI 58.8, 72.8) with QM dosing in the group with high TGs and normal HDL-C, 59.2% (95% CI 50.2, 68.2) with Q2W dosing and 53.4% (95% CI 49.3, 57.6) with QM dosing in the group with high TGs and low HDL-C, and 63.9% (95% CI 56.1, 71.7) with Q2W dosing and 55.2% (95% CI 50.1, 60.3) with QM dosing in the group with normal TGs and normal HDL-C, all *P*-values < 0.0001.

Significantly greater reductions with evolocumab treatment than placebo were also observed at week 12 for ApoB, and at the mean of weeks 10 and 12 for Lp(a), VLDL-C, remnant cholesterol, and TGs in individuals with and without high TGs (evolocumab Q2W and QM combined for VLDL-C, remnant cholesterol, and TGs; all *P*-values < 0.0001 except for VLDL-C in the group with high TGs and normal HDL-C, P = 0.0002). For HDL-C, small, but significant, treatment differences favoring evolocumab were observed in patients with and without high TGs.

The analyses of percentage change from baseline with evolocumab treatment in LDL-C and non-HDL-C to the mean of weeks 10 and 12, and in total ApoB at week 12 were consistent between those receiving insulin (total n = 352) and those not receiving insulin therapy (total n = 751) across all 3 groups (data not shown).

#### Change from baseline

Significant treatment group differences were observed in the improvement from baseline to the mean of weeks 10 and 12 across all lipid parameters (Additional file 1: Table S3). At the mean of weeks 10 and 12, compared with placebo, evolocumab treatment reduced LDL-C by an LS mean of 1.56 mmol/L (95% CI 1.06, 2.05) with Q2W dosing and 1.98 mmol/L (95% CI 1.71, 2.24) with QM dosing in the group with high TGs and normal HDL-C, 1.74 mmol/L (95% CI 1.45, 2.02) with Q2W dosing and 1.66 mmol/L (95% CI 1.49, 1.83) with QM dosing in the group with high TGs and low HDL-C, and 1.62 mmol/L (95% CI 1.38, 1.87) with Q2W dosing and 1.57 mmol/L (95% CI 1.40, 1.73) with QM dosing in the group with normal TGs and normal HDL-C, all *P*-values < 0.0001. Compared with placebo, evolocumab treatment reduced non-HDL-C by an LS mean of 1.70 mmol/L (95% CI 1.16, 2.23) with Q2W dosing and 2.47 mmol/L (95% CI 2.16, 2.78) with QM dosing in the group with high TGs and normal HDL-C, 2.14 mmol/L (95% CI 1.76, 2.53) with Q2W dosing and 1.98 mmol/L (95% CI 1.79, 2.18) with QM dosing in the group with high TGs and low HDL-C, and 1.78 mmol/L (95% CI 1.52, 2.04) with Q2W dosing and 1.67  mmol/L (95% CI 1.49, 1.84) with QM dosing in the group with normal TGs and normal HDL-C, all *P*-values < 0.0001.

At week 12, compared with placebo, evolocumab treatment reduced ApoB by an LS mean of 0.42 g/L (95% CI 0.27, 0.56) with Q2W dosing and 0.55 g/L (95% CI 0.48, 0.62) with QM dosing in the group with high TGs and normal HDL-C, 0.56 g/L (95% CI 0.46, 0.66) with Q2W dosing and 0.40 g/L (95% CI 0.34, 0.45) with QM dosing in the group with high TGs and low HDL-C, and 0.45 g/L (95% CI 0.38, 0.52) with Q2W dosing and 0.36 g/L (95% CI 0.31, 0.40) with QM dosing in the group with normal TGs and normal HDL-C, all *P*-values < 0.0001.

Greater reductions from baseline for evolocumab than placebo were also observed at the mean of weeks 10 and 12 in all groups for Lp(a), VLDL-C, remnant cholesterol and TGs (all *P*-values < 0.0001 except for VLDL-C in the group with high TGs and normal HDL-C, P = 0.0002). For HDL-C, small, but significant mean treatment differences favoring evolocumab were observed in patients with high TGs, except in the normal TGs and normal HDL-C group with Q2W dosing even though there were similar mean absolute changes from baseline in the evolocumab Q2W and QM groups.

#### Correlation of changes in ApoB100 with non-HDL-C, remnant cholesterol and TGs

**Fig. 3:**
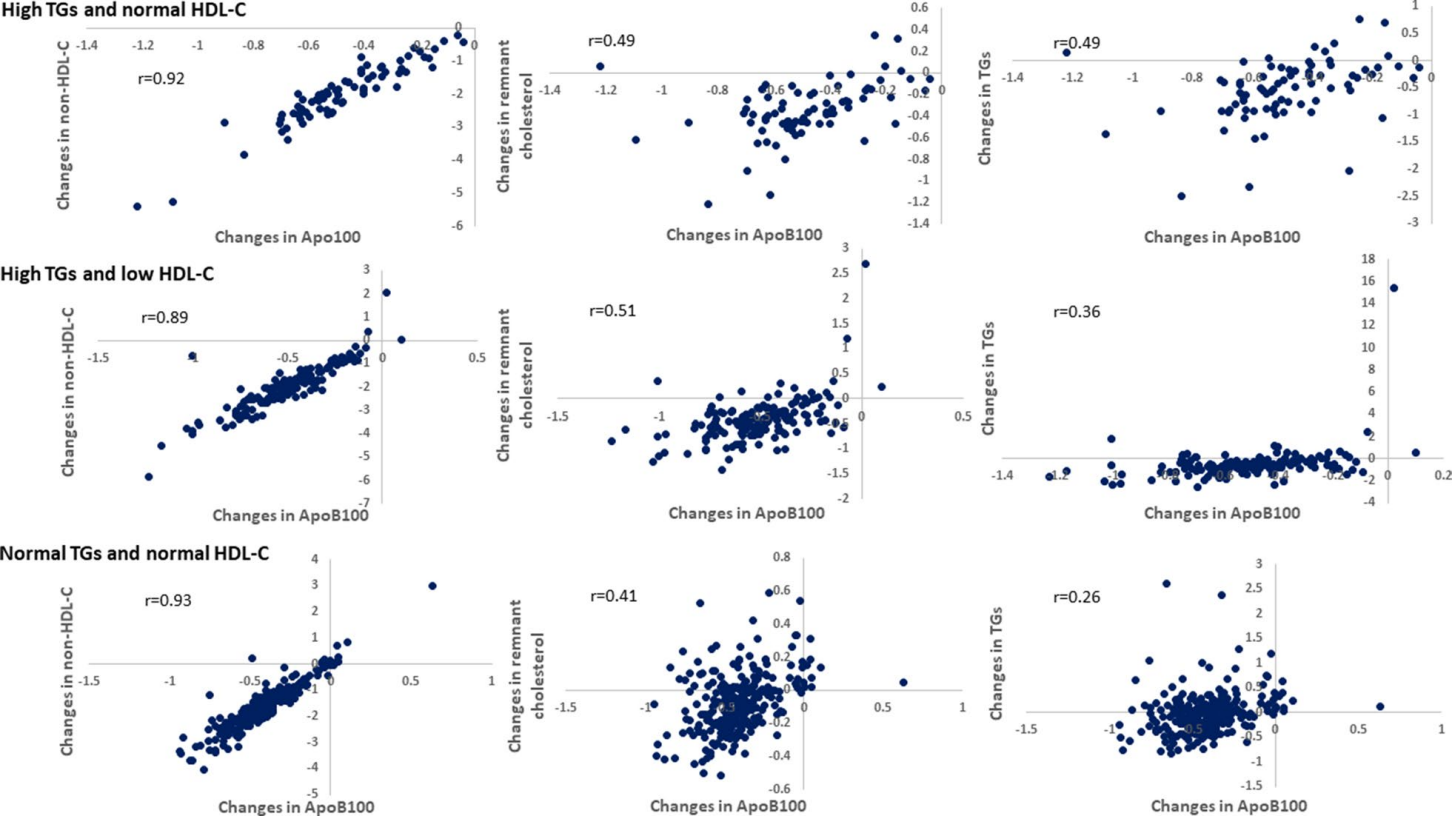
Correlation of changes in ApoB100 with non-HDL-C, remnant cholesterol and TGs. The X axis represents changes in ApoB100 from baseline to the mean of weeks 10 and 12 in evolocumab-treated patients; the Y axis represents changes in non-HDL-C, TGs or remnant cholesterol from baseline to the mean of weeks 10 and 12 in evolocumab-treated patients. All correlation coefficients are statistically significant at P < 0.0001. *ApoB* apolipoprotein B, *HDL-C* high-density lipoprotein cholesterol, *LDL-C* low-density lipoprotein cholesterol, *non-HDL-C* non-high-density lipoprotein cholesterol, *TG* triglycerides

In the evolocumab-treated individuals with Q2W and QM dosing combined, changes in ApoB100 from baseline to the mean of weeks 10 and 12 were strongly correlated with changes in non-HDL-C from baseline to the mean of weeks 10 and 12 (overall r = 0.93, P < 0.0001). The strong correlation existed in all three subgroups: the group with high TGs and normal HDL-C (r = 0.92, P < 0.0001); the group with high TGs and low HDL-C (r = 0.89, P < 0.0001); the group with normal TGs and normal HDL-C (r = 0.93, P < 0.0001) (Fig. 3). The changes in ApoB100 from baseline to the mean of weeks 10 and 12 were moderately correlated with changes in remnant cholesterol (overall r = 0.45, P < 0.0001). The moderate correlations persisted in all three subgroups: the group with high TGs and normal HDL-C (r = 0.49, P < 0.0001); the group with high TGs and low HDL-C (r = 0.51, P < 0.0001); the group with normal TGs and normal HDL-C (r = 0.41, P < 0.0001). The correlation with TGs was moderate in the group with high TGs and normal HDL-C (r = 0.49, P < 0.0001), and weaker in the other two groups (Fig. 3).

#### Goal attainment

**Fig. 4:**
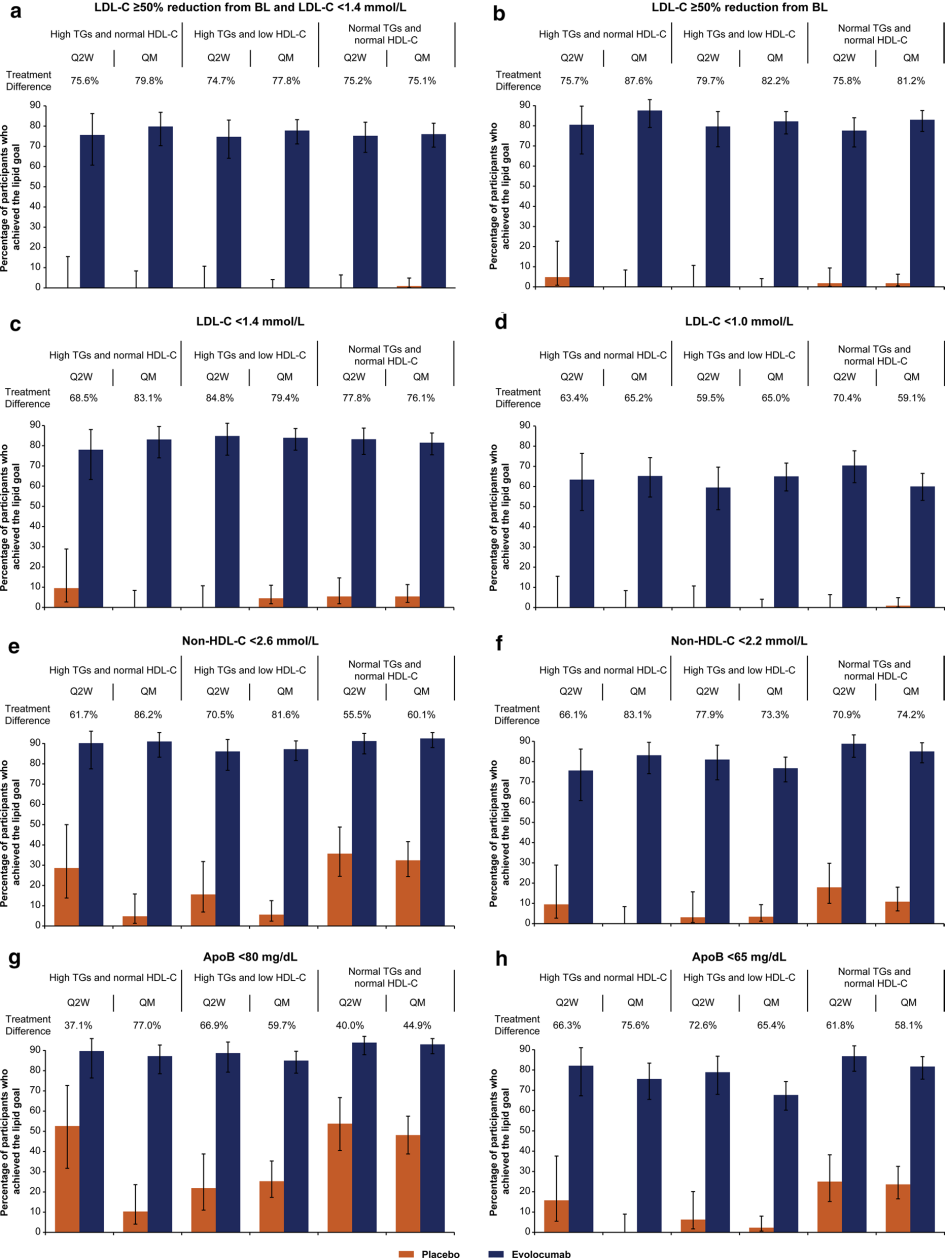
Percentage of participants who achieved each lipid treatment goal with evolocumab vs placebo on background statin therapy. Values shown are means of weeks 10 and 12 except for ApoB, which was measured at week 12. The treatment differences between evolocumab and placebo for all endpoints are statistically significant at *P* < 0.0001 based on Cochran-Mantel–Haenszel tests stratified by study. When the calculated LDL-C was < 1.0 mmol/L or TGs were > 4.5 mmol/L, calculated LDL-C was replaced with ultracentrifugation to inform LDL-C and VLDL-C from the same blood sample, if available. *ApoB* apolipoprotein B, *BL* baseline, *LDL-C* low-density lipoprotein cholesterol, *non-HDL-C* non-high-density lipoprotein cholesterol, *Q2W* every 2 weeks, *QM* monthly, TGs triglycerides

The differences in the percentages of individuals who attained the 2019 ESC/EAS lipid treatment goals in the evolocumab-treated vs placebo-treated groups were substantial and significant for all goals measured (Fig. 4 and Additional file 1: Table S4). For the goal combining 50% LDL-C reduction plus attainment of LDL-C < 1.4 mmol/L at the mean of weeks 10 and 12, more evolocumab- than placebo-treated individuals were able to meet the goal in the high TGs and normal HDL-C group (treatment difference [evolocumab minus placebo]: Q2W, 75.6%; QM, 79.8%), the high TGs and low HDL-C group (treatment difference [evolocumab minus placebo]: Q2W, 74.7%; QM, 77.8%), and the normal group (treatment difference [evolocumab minus placebo]: Q2W, 75.2%; QM, 75.1%), all *P*-values < 0.0001. Similarly, for the LDL-C goals of at least 50% response, LDL-C < 1.4 mmol/L, and LDL-C < 1.0 mmol/L, a greater percentage of evolocumab- than placebo-treated individuals attained each goal for all three groups and dosing regimens, all *P*-values < 0.0001.

For the goals of reduction of non-HDL-C to < 2.6 mmol/L and < 2.2 mmol/L at the mean of weeks 10 and 12, a greater percentage of individuals treated with evolocumab than placebo attained the goals in each group and each dosing regimen, (all *P*-values < 0.0001). Similarly, for the goals of reduction of ApoB to < 80 mg/dL and < 65 mg/dL at week 12, a greater percentage of individuals treated with evolocumab than placebo attained the goals, regardless of the group or dosing regimen (all *P*-values < 0.0001).

## Discussion

This pooled analysis of data from two phase 3, dedicated T2DM studies demonstrated that both dosing regimens of evolocumab consistently and effectively reduced ApoB and the cholesterol levels from ApoB-containing lipoproteins, as reflected by the reduction in non-HDL-C levels, across individuals with high TGs with and without AD.

AD increases CVD risk and is a common condition in patients with T2DM. In this analysis, approximately half of individuals with T2DM and elevated LDL-C had high TGs at baseline. Among these individuals, 66.5% had AD (high TGs and low HDL-C). As expected, baseline non-HDL-C, ApoB, VLDL-C, and remnant cholesterol were higher in the groups of participants with high TGs than in the group with normal TGs. At the same time, among individuals with high TGs, baseline LDL-C was lower in the group with AD.

### Reduction in ApoB and cholesterol carried by ApoB-containing lipoproteins

In individuals with high TGs with and without AD treated with at least moderate-intensity statin, the addition of evolocumab (Q2W or QM), compared to placebo, significantly reduced mean LDL-C by 66.7% to 74.3%, non-HDL-C by 53.4% to 65.8%, and ApoB by 41.5% to 56.6% (all *P*-values < 0.0001). Results were similar in the group of individuals with normal TGs. This is the first analysis reporting the effect of evolocumab in individuals with high TGs with and without AD. The results are consistent with prior evolocumab studies in primary hyperlipidemia [33, 34] and in type 2 diabetes from the BANTING [25] and BERSON [24] studies. While the BANTING and BERSON studies did not measure LDL particle (LDL-P) number and size, Toth et al. have previously shown that in a broad range of patients with hyperlipidemia, treatment with evolocumab reduced both large and small LDL-P. The percentage reduction in large LDL-P was approximately twice that in small LDL-P, which may manifest less efficient clearance of small, dense LDL-P by the LDL-R. The reported percentage reduction in total LPL-P and small LDL-P in the subset of patients with T2D were similar to the overall population [35]. In the open-label ODYSSEY DM-DYSLIPIDEMIA study (NCT02642159) in participants with T2DM and mixed dyslipidemia, alirocumab, another PCSK9i, significantly reduced all three lipid parameters (LDL-C, non-HDL-C, and ApoB) and LDL-P number when compared with usual care.

We found a robust correlation between ApoB100 and non-HDL-C with evolocumab treatment. This observation is consistent with prior reports [36, 37] and clinically meaningful because both non-HDL-C and ApoB are considered secondary goals to adjust further or intensify the lipid-lowering therapy [5, 20]. Of note, non-HDL-C can be easily calculated from the routine lipid panel (TC minus HDL-C) with no added cost. Both ApoB and non-HDL-C are also recommended to improve ASCVD risk assessment, particularly in situations where discordance between these measures and LDL-C may occur (e.g. diabetes mellitus, obesity, metabolic syndrome, high TGs, and very low LDL-C levels). The moderate positive correlation of ApoB100 with remnant cholesterol and the weaker correlation with TGs may be explained by the fact that most ApoB100 is from LDL particles. In contrast, remnant cholesterol and TGs represent the content from TRL (excludes LDL).

We have also investigated the associations between evolocumab treatment and Lp(a) across all three groups. Because a positive correlation has been reported between higher Lp(a) levels and the development of ASCVD, and about 90% of Lp(a) levels are inherited, the 2019 ESC/EAS guidelines recommend that its measurement should be considered in each person’s lifetime [5]. Statins often increase Lp(a) levels [38], which is aligned with the changes observed in the placebo groups. In our high-risk patient population with high TGs, with a median Lp(a) at baseline that ranged from 21.0 nmol/L to 37.0 nmol/L, evolocumab was associated with a significant reduction of Lp(a), by a median of 37.5% to 53.9%.

Our analyses’ findings are important because reducing ApoB levels and the cholesterol content across all the spectrum of ApoB lipoproteins (in addition to LDL-C) represents an opportunity to address the residual ASCVD risk that still exists in many patients with T2DM despite normal LDL-C levels and receiving optimal statin therapy [20]. Recently, an analysis performed from a Danish contemporary population-based cohort excluding individuals with a history of myocardial infarction at baseline and receiving lipid-lowering therapy, the hazard ratio for myocardial infarction per a 100 mg/dL higher concentration of ApoB was 2.21 (95% CI 1.90 to 2.58) [39].

### Triglycerides and cholesterol content on TRL and their remnants

Remnant lipoproteins (chylomicron remnants, VLDL, and IDL) are smaller cholesterol-enriched particles that, via intimal deposition of cholesterol, may contribute to atherogenesis and the residual ACVD risk [40]. A simple way to estimate remnant cholesterol levels is by subtracting LDL-C and HDL-C to total cholesterol. In individuals with high TGs with and without AD receiving statin therapy, remnant cholesterol levels, which are commonly elevated in patients with T2DM and are a maker of elevated TGs in plasma, were significantly reduced with evolocumab by a median of 28.9% to 34.2% compared with placebo (all *P*-values < 0.0001). VLDL-C and TGs levels were also significantly reduced with evolocumab in individuals with high TGs with and without AD compared to placebo (all *P*-values < 0.0001 except for VLDL-C in the group with high TGs and normal HDL-C, P = 0.0002). The reductions in remnant cholesterol, VLDL-C, and TGs in individuals with normal TGs were also significant, albeit the magnitude of the percentage change from baseline was clearly lower compared with individuals with high TGs. This is expected as the baseline levels of these lipid parameters were lower than those with high TGs. Interestingly, Sharma et al. demonstrated that in individuals with mixed dyslipidemia, higher baseline VLDL-C levels were associated with a greater percentage decrease in TGs following 12 weeks of lipid-lowering therapy [41]. The higher percentage reduction in non-HDL-C and ApoB when compared with VLDL-C and remnant cholesterol is expected because the calculation of non-HDL-C and the measurement of ApoB levels also account for LDL-C, and Lp(a) and LDL particles, respectively.

The significant improvement in the lipid profile (LDL-C, non-HDL-C, ApoB, VLDL-C, remnant cholesterol, and TGs) of individuals with T2DM with or without AD treated with evolocumab could be explained by the increase in the LDL receptor (LDLR) expression in the liver, which recognizes both ApoB100 on LDL and ApoE on VLDL and IDL. A recent study [42] performed in individuals with T2D showed a significant increase in the fractional catabolic rate (FCR) for smaller VLDL-apoB100 and -triglyceride and a 54% increase in the overall FCR of IDL with 12 weeks of treatment with evolocumab. Of note, ApoE is also a ligand for low-density lipoprotein receptor-related protein 1 (LRP1). Since in vitro studies [43, 44] have shown the ability of PCSK9 to also modulate the expression in the liver of VLDL receptor, LRP1, and ApoE receptor, we cannot completely rule out an effect of PCSK9 inhibition on these metabolic pathways for the observed reduction of VLDL-C and IDL-C.

### Lipid treatment goals attainment

The participants in this analysis represent a population in which a vast majority had a very high risk of CVD events. For such individuals, the ESC/EAS 2019 dyslipidemia guidelines recommend an LDL-C goal of < 1.4 mmol/L, at least a 50% reduction from baseline in LDL-C, and secondary goals of non-HDL-C < 2.2 mmol/L and ApoB < 65 mg/dL. Similar aggressive goals are recommended by the American Association of Clinical Endocrinologists and the American College of Endocrinology [20] in patients with diabetes and ASCVD. Our analysis shows that the recommended goals for high-risk patients are attainable when a PCSK9i is added to background statin therapy. Evolocumab enabled the majority of individuals with and without high TGs (74.7% to 79.8%) to achieve both an LDL-C goal of < 1.4 mmol/L and an LDL-C reduction of at least 50%. More than 68% achieved each separately. By contrast, in a recent Italian registry [45], only 3.2% of 4751 patients with very high CVD risk attained the combined LDL-C goal, despite 55% of patients receiving high-intensity statins (4.8% received ezetimibe). The DA VINCI study [46], a cross-sectional study that collected data from 18 European countries, showed that less than 30% of patients at high- or very high-risk receiving high-intensity statin monotherapy or a statin in combination with ezetimibe attained their 2019 ESC/EAS risk-based LDL-C goal. Notably, an LDL-C < 1.0 mmol/L, which may be considered for patients with a recurrent vascular event within 2 years, was achieved by 59.1% to 70.4% of individuals with and without high TGs who received evolocumab, compared with placebo (only one individual reached an LDL-C of less than 1.4 mmol/L), on top of statin therapy. In addition, more than 75% of evolocumab-treated individuals achieved the non-HDL-C goal of less than 2.2 mmol/L compared with less than 18% of placebo-treated individuals. ApoB less than 65 mg/dL was achieved by more than 67% of individuals at week 12 compared with 25% or less of placebo-treated individuals.

Limitations include the post hoc analysis of the data and the short duration of the two studies. Also, most of the participants were Caucasians and Asians, with only a minority identified as Hispanic/Latino or Black or African American.

## Conclusions

In conclusion, in individuals with T2DM with or without AD receiving background statin therapy, evolocumab significantly reduced LDL-C, non-HDL-C, ApoB, Lp(a), VLDL-C, and remnant cholesterol. Also, evolocumab Q2W or QM enabled a majority of individuals at high/very-high cardiovascular disease risk to achieve the LDL-C, non-HDL-C, and ApoB goals recommended by the 2019 ESC/EAS dyslipidemia guidelines.

## Supplementary Information

**Additional file 1: Table S1.** Patient demographic and disease characteristics at baseline by treatment group and dosing regimen. **Table S2**. Percentage change from baseline lipid values with evolocumab vs placebo treatment (mean of weeks 10 and 12). **Table S3.** Change from baseline lipid values with evolocumab vs placebo treatment (mean of weeks 10 and 12). **Table S4.** Percentage of patients who met each lipid goal with evolocumab vs placebo treatment (mean of weeks 10 and 12).

## Data Availability

Qualified researchers may request data from Amgen clinical studies. Complete details are available at https://wwwext.amgen.com/science/clinical-trials/clinical-data-transparency-practices/clinical-trial-data-sharing-request.

## Abbreviations

AD: Atherogenic dyslipidemia
ApoB: Apolipoprotein B
ASCVD: Atherosclerotic cardiovascular disease
CI: Confidence interval
CVD: Cardiovascular disease
EAS: European Atherosclerosis Society
ESC: European Society of Cardiology
FCR: Fractional catabolic rate
HDL: High-density lipoprotein
IDL: Intermediate-density lipoprotein
LDL: Low-density lipoprotein
LDL-P: LDL particle
LDLR: LDL receptor
Lp(a): Lipoprotein(a)
LRP1: Lipoprotein receptor-related protein 1
LS: Least squares
PCSK9: Proprotein convertase subtilisin/kexin type 9 inhibitor
Q2W: Every 2 weeks
QM: Monthly
SC: Subcutaneous
T2DM: Type 2 diabetes mellitus
TG: Triglycerides
TRL: Triglyceride-rich lipoprotein
VLDL: Very-low-density lipoprotein
