# Supplementary material for: Effects of evolocumab in individuals with type 2 diabetes with and without atherogenic dyslipidemia: An analysis from BANTING and BERSON
Source: Cardiovasc Diabetol. 2021 Apr 30;20:94. doi: 10.1186/s12933-021-01287-6 (PMC8091704; doi:10.1186/s12933-021-01287-6)
Supplement: Supplementary file 1 — Additional file 1: Table S1. Patient demographic and disease characteristics at baseline by treatment group and dosing regimen. Table S2. Percentage change from baseline lipid values with evolocumab vs placebo treatment (mean of weeks 10 and 12). Table S3. Change from baseline lipid values with evolocumab vs placebo treatment (mean of weeks 10 and 12). Table S4. Percentage of patients who met each lipid goal with evolocumab vs placebo treatment (mean of weeks 10 and 12). [file 12933_2021_1287_MOESM1_ESM.docx]

**Supplemental Tables**

**Supplemental Table 1. Patient demographic and disease characteristics at baseline by treatment group and dosing regimen**

| **Characteristic** | **High TGs and normal HDL-C** | | | | **High TGs and low HDL-C** | | | | **Normal TGs and normal HDL-C** | | | |
| --- | --- | --- | --- | --- | --- | --- | --- | --- | --- | --- | --- | --- |
|  | **Q2W** | | **QM** | | **Q2W** | | **QM** | | **Q2W** | | **QM** | |
|  | **Placebo** | **Evolocumab** | **Placebo** | **Evolocumab** | **Placebo** | **Evolocumab** | **Placebo** | **Evolocumab** | **Placebo** | **Evolocumab** | **Placebo** | **Evolocumab** |
|  | **(N=21)** | **(N=42)** | **(N=43)** | **(N=90)** | **(N=33)** | **(N=81)** | **(N=92)** | **(N=183)** | **(N=62)** | **(N=129)** | **(N=116)** | **(N=212)** |
| Sex, female, n (%) | 10 (47.6) | 18 (42.9) | 18 (41.9) | 40 (44.4) | 23 (69.7) | 53 (65.4) | 49 (53.3) | 90 (49.2) | 37 (59.7) | 62 (48.1) | 58 (50.0) | 99 (46.7) |
| Age, years, mean (SD) | 60.1 (9.0) | 60.0 (8.6) | 64.3 (8.1) | 62.8 (8.6) | 60.2 (9.3) | 58.6 (9.1) | 60.1 (7.8) | 62.0 (8.0) | 62.8 (8.2) | 62.3 (8.1) | 62.5 (9.2) | 62.1 (8.1) |
| Ethnicity  Hispanic/Latino, n (%) | 3 (14.3) | 7 (16.7) | 9 (20.9) | 21 (23.3) | 4 (12.1) | 20 (24.7) | 25 (27.2) | 40 (21.9) | 9 (14.5) | 22 (17.1) | 14 (12.1) | 33 (15.6) |
| Race, n (%) |  |  |  |  |  |  |  |  |  |  |  |  |
| White | 12 (57.1) | 19 (45.2) | 28 (65.1) | 63 (70.0) | 17 (51.5) | 41 (50.6) | 62 (67.4) | 124 (67.8) | 29 (46.8) | 50 (38.8) | 54 (46.6) | 107 (50.5) |
| Asian | 9 (42.9) | 20 (47.6) | 6 (14.0) | 20 (22.2) | 15 (45.5) | 34 (42.0) | 17 (18.5) | 39 (21.3) | 30 (48.4) | 72 (55.8) | 42 (36.2) | 72 (34.0) |
| Black or African American | 0 (0.0) | 1 (2.4) | 7 (16.3) | 7 (7.8) | 1 (3.0) | 3 (3.7) | 5 (5.4) | 8 (4.4) | 1 (1.6) | 3 (2.3) | 19 (16.4) | 28 (13.2) |
| American Indian or Alaska native | 0 (0.0) | 1 (2.4) | 1 (2.3) | 0 (0.0) | 0 (0.0) | 0 (0.0) | 6 (6.5) | 6 (3.3) | 0 (0.0) | 0 (0.0) | 0 (0.0) | 2 (0.9) |
| Native Hawaiian or other Pacific Islander | 0 (0.0) | 0 (0.0) | 0 (0.0) | 0 (0.0) | 0 (0.0) | 0 (0.0) | 0 (0.0) | 1 (0.5) | 0 (0.0) | 0 (0.0) | 0 (0.0) | 0 (0.0) |
| Multiple | 0 (0.0) | 1 (2.4) | 1 (2.3) | 0 (0.0) | 0 (0.0) | 3 (3.7) | 2 (2.2) | 4 (2.2) | 2 (3.2) | 4 (3.1) | 1 (0.9) | 2 (0.9) |
| Other | 0 (0.0) | 0 (0.0) | 0 (0.0) | 0 (0.0) | 0 (0.0) | 0 (0.0) | 0 (0.0) | 1 (0.5) | 0 (0.0) | 0 (0.0) | 0 (0.0) | 1 (0.5) |
| BMI, kg/m^2^, mean (SD) | 30.1 (6.3) | 28.5 (4.8) | 31.7 (7.9) | 31.0 (6.3) | 29.1 (5.5) | 30.1 (5.4) | 32.0 (6.9) | 32.3 (6.6) | 27.9 (6.1) | 26.9 (3.7) | 29.2 (6.3) | 29.3 (5.7) |
| Waist circumference, cm, mean (SD) | 98.8 (15.4) | 98.4 (10.7) | 106.4 (17.6) | 105.4 (15.4) | 98.6 (15.7) | 100.1 (12.6) | 106.1 (16.5) | 106.0 (16.6) | 94.3 (12.6) | 94.1 (10.0) | 98.2 (15.2) | 99.2 (14.0) |
| Duration of diabetes, years, mean (SD) | 9.104 (9.339) | 9.916 (6.779) | 9.842 (6.545) | 10.856 (8.242) | 7.343 (5.613) | 8.776 (5.928) | 10.878 (7.917) | 11.325 (7.559) | 11.077 (8.963) | 9.108 (6.587) | 9.633 (7.002) | 10.888 (8.093) |
| Fasting serum glucose, mmol/L, median (Q1, Q3) | 7.70 (6.20, 8.60) | 8.05 (6.70, 10.90) | 7.10 (6.00, 8.90) | 7.10 (6.10, 8.90) | 7.00 (6.00, 9.20) | 7.80 (6.30, 10.30) | 7.90 (6.20, 10.20) | 8.30 (6.40, 10.70) | 6.80 (5.70, 8.10) | 7.20 (6.40, 8.60) | 6.80 (5.95, 8.60) | 6.90 (5.70, 8.70) |
| HbA1c (fraction of 1), median (Q1, Q3) | 0.0710 (0.0620, 0.0780) | 0.0695  (0.0650, 0.0860) | 0.0680  (0.0640, 0.0750) | 0.0700  (0.0640, 0.0800) | 0.0670 (0.0590, 0.0790) | 0.0710 (0.0620, 0.0860) | 0.0780 (0.0675, 0.0880) | 0.0750 (0.0670, 0.0840) | 0.0660 (0.0600, 0.0730) | 0.0700 (0.0650, 0.0790) | 0.0680 (0.0625, 0.0780) | 0.0700 (0.0620, 0.0815) |
| Cardiovascular risk category per 2019 ESC/EAS^1^ guidelines |  |  |  |  |  |  |  |  |  |  |  |  |
| Very high risk, n (%) | 21 (100.0) | 39 (92.9) | 37 (86.0) | 85 (94.4) | 32 (97.0) | 74 (91.4) | 84 (91.3) | 169 (92.3) | 56 (90.3) | 114 (88.4) | 98 (84.5) | 189 (89.2) |
| High risk, n (%) | 0 (0.0) | 3 (7.1) | 6 (14.0) | 5 (5.6) | 1 (3.0) | 7 (8.6) | 8 (8.7) | 14 (7.7) | 6 (9.7) | 15 (11.6) | 18 (15.5) | 23 (10.8) |
| Statin use per 2018 ACC/AHA guidelines^2^ |  |  |  |  |  |  |  |  |  |  |  |  |
| Any statin use^3^, n (%) | 13 (61.9) | 28 (66.7) | 35 (81.4) | 76 (84.4) | 19 (57.6) | 57 (70.4) | 76 (82.6) | 156 (85.2) | 34 (54.8) | 65 (50.4) | 86 (74.1) | 151 (71.2) |
| High-intensity statin, n (%) | 0 (0.0) | 0 (0.0) | 12 (27.9) | 29 (32.2) | 1 (3.0) | 9 (11.1) | 34 (37.0) | 58 (31.7) | 3 (4.8) | 2 (1.6) | 20 (17.2) | 43 (20.3) |
| Moderate-intensity statin, n (%) | 12 (57.1) | 25 (59.5) | 23 (53.5) | 46 (51.1) | 17 (51.5) | 42 (51.9) | 39 (42.4) | 92 (50.3) | 28 (45.2) | 60 (46.5) | 65 (56.0) | 102 (48.1) |
| Ezetimibe, n (%) | 0 (0.0) | 1 (2.4) | 1 (2.3) | 3 (3.3) | 0 (0.0) | 2 (2.5) | 4 (4.3) | 5 (2.7) | 1 (1.6) | 1 (0.8) | 4 (3.4) | 8 (3.8) |
| Lipid concentration (IUs) |  |  |  |  |  |  |  |  |  |  |  |  |
| LDL-C, mmol/L, mean (SD) | 2.636 (0.761) | 2.568 (1.157) | 2.897 (0.959) | 2.815 (0.866) | 2.514 (0.860) | 2.589 (0.939) | 2.507 (0.806) | 2.604 (0.877) | 2.391 (0.963) | 2.371 (0.760) | 2.619 (0.833) | 2.596 (0.841) |
| Non-HDL-C, mmol/L, mean (SD) | 3.617 (0.794) | 3.566 (1.157) | 3.918 (1.050) | 3.838 (0.918) | 3.671 (1.132) | 3.733 (0.981) | 3.738 (0.857) | 3.820 (0.988) | 2.899 (0.998) | 2.897 (0.800) | 3.133 (0.879) | 3.115 (0.870) |
| Total ApoB, g/L, mean (SD) | 0.949 (0.204) | 0.938 (0.256) | 1.003 (0.234) | 0.991 (0.215) | 0.962 (0.276) | 1.004 (0.247) | 0.990 (0.224) | 0.992 (0.237) | 0.788 (0.222) | 0.799 (0.180) | 0.844 (0.213) | 0.844 (0.218) |
| Lp(a), nmol/L |  |  |  |  |  |  |  |  |  |  |  |  |
| Mean (SD) | 44.5  (64.8) | 92.9  (124.2) | 70.8 (114.3) | 86.1  (109.1) | 85.6 (128.7) | 63.0  (90.8) | 75.6 (93.3) | 64.9  (98.3) | 76.3  (91.9) | 59.3  (78.6) | 93.8 (112.5) | 91.3  (111.4) |
| Median (Q1, Q3) | 22.0 (10.0, 42.0) | 31.5 (6.0, 150.0) | 18.0 (7.0, 76.0) | 43.5 (12.0, 126.0) | 25.0 (14.0, 64.0) | 20.0 (9.0, 76.0) | 29.5 (7.0, 138.0) | 22.5 (7.0, 77.0) | 43.0 (12.0, 101.0) | 25.0 (11.0, 76.0) | 49.0 (12.0, 138.0) | 41.0 (13.0, 137.0) |
| VLDL-C , mmol/L, |  |  |  |  |  |  |  |  |  |  |  |  |
| Mean (SD) | 0.996  (0.211) | 1.022  (0.258) | 1.013  (0.194) | 1.016  (0.232) | 1.093 (0.301) | 1.147 (0.263) | 1.155 (0.329) | 1.178 (0.335) | 0.507 (0.132) | 0.526 (0.143) | 0.516 (0.129) | 0.519 (0.129) |
| Median (Q1, Q3) | 0.850  (0.830, 1.220) | 0.980  (0.830, 1.140) | 0.975  (0.880, 1.090) | 0.948  (0.850, 1.090) | 0.980 (0.830, 1.270) | 1.090 (0.910, 1.350) | 1.060 (0.895, 1.350) | 1.090 (0.895, 1.370) | 0.490 (0.410, 0.600) | 0.540 (0.410, 0.650) | 0.525 (0.415, 0.605) | 0.520 (0.425, 0.623) |
| Remnant cholesterol, mmol/L |  |  |  |  |  |  |  |  |  |  |  |  |
| Mean (SD) | 0.997  (0.209) | 0.997  (0.192) | 1.018  (0.231) | 1.022  (0.251) | 1.156 (0.459) | 1.143 (0.260) | 1.225 (0.468) | 1.217 (0.430) | 0.507 (0.131) | 0.525 (0.142) | 0.515 (0.131) | 0.519 (0.129) |
| Median (Q1, Q3) | 0.860  (0.820, 1.220) | 0.985  (0.830, 1.140) | 0.970  (0.850, 1.090) | 0.945  (0.850, 1.120) | 0.990 (0.830, 1.270) | 1.090 (0.910, 1.350) | 1.073 (0.910, 1.435) | 1.115 (0.900, 1.380) | 0.495 (0.410, 0.590) | 0.540 (0.410, 0.650) | 0.523 (0.420, 0.603) | 0.520 (0.430, 0.630) |
| TGs, mmol/L |  |  |  |  |  |  |  |  |  |  |  |  |
| Mean (SD) | 2.269  (0.735) | 2.234  (0.561) | 2.287  (0.638) | 2.243  (0.562) | 2.556 (1.120) | 2.540 (0.632) | 2.859 (2.158) | 2.740 (1.121) | 1.107 (0.280) | 1.150 (0.312) | 1.126 (0.281) | 1.131 (0.283) |
| Median (Q1, Q3) | 1.890  (1.800, 2.640) | 2.145  (1.820, 2.460) | 2.145  (1.910, 2.445) | 2.055  (1.840, 2.430) | 2.150 (1.820, 2.780) | 2.430 (1.990, 2.960) | 2.355 (1.965, 3.195) | 2.470 (1.970, 3.120) | 1.100 (0.920, 1.280) | 1.180 (0.890, 1.410) | 1.143 (0.913, 1.323) | 1.130 (0.923, 1.380) |
| HDL-C, mmol/L, mean (SD) | 1.392  (0.257) | 1.383  (0.200) | 1.333  (0.239) | 1.324  (0.207) | 0.965 (0.160) | 0.952 (0.189) | 0.941 (0.164) | 0.927 (0.183) | 1.441 (0.285) | 1.414 (0.244) | 1.513 (0.361) | 1.456 (0.308) |

^1^Mach F, Baigent C, Catapano AL, Koskinas KC, Casula M, Badimon L, et al. 2019 ESC/EAS Guidelines for the management of dyslipidaemias: lipid modification to reduce cardiovascular risk. Eur Heart J. 2020;41:111-88.

^2^Grundy SM, Stone NJ, Bailey AL, Beam C, Birtcher KK, Blumenthal RS, et al. 2018 AHA/ACC/AACVPR/AAPA/ABC/ACPM/ADA/AGS/APhA/ASPC/NLA/ PCNA Guideline on the management of blood cholesterol: a report of the American College of Cardiology/American Heart Association Task Force on Clinical Practice Guidelines. Circulation. 2019;139:e1082-e1143.

When the calculated LDL-C was <1.0 mmol/L or TGs were >4.5 mmol/L, calculated LDL-C was replaced with ultracentrifugation to inform LDL-C and VLDL-C from the same blood sample, if available.

^3^Represents use of statin therapy before initiating any treatment intervention (including atorvastatin and/or study drug); in BANTING, 99.3% of patients were on moderate- or high-intensity statin at randomization; in BERSON, all patients initiated moderate-intensity atorvastatin at enrollment.

ACC = American College of Cardiology; AHA = American Heart Association; ApoB = apolipoprotein B; BMI = body mass index; EAS = European Atherosclerosis Society; ESC = European Society of Cardiology; HbA1c = hemoglobin A1c; HDL-C = high-density lipoprotein cholesterol; IU = international unit; LDL-C = low-density lipoprotein cholesterol; Lp(a) = lipoprotein(a); non-HDL-C = non-high-density lipoprotein cholesterol; Q = quartile; Q2W = every 2 weeks; QM = monthly; SD = standard deviation; TGs = triglycerides; VLDL-C = very low-density lipoprotein cholesterol.

**Supplemental Table 2. Percentage change from baseline lipid values with evolocumab vs placebo treatment (mean of weeks 10 and 12)**

**Supplemental Table 2A:**

| **Lipid parameters, percent change from baseline values** | **High TGs and normal HDL-C** | | | | **High TGs and low HDL-C** | | | | **Normal TGs and normal HDL-C** | | | |
| --- | --- | --- | --- | --- | --- | --- | --- | --- | --- | --- | --- | --- |
|  | **Q2W** | | **QM** | | **Q2W** | | **QM** | | **Q2W** | | **QM** | |
|  | **Placebo** | **Evolocumab** | **Placebo** | **Evolocumab** | **Placebo** | **Evolocumab** | **Placebo** | **Evolocumab** | **Placebo** | **Evolocumab** | **Placebo** | **Evolocumab** |
|  | **(N=21)** | **(N=42)** | **(N=43)** | **(N=90)** | **(N=33)** | **(N=81)** | **(N=92)** | **(N=183)** | **(N=62)** | **(N=129)** | **(N=116)** | **(N=212)** |
| **LDL-C** |  |  |  |  |  |  |  |  |  |  |  |  |
| n | 21 | 41 | 42 | 89 | 32 | 79 | 89 | 180 | 56 | 125 | 111 | 200 |
| LS mean estimate (SE) | -2.00 (4.99) | -62.87 (3.57) | 5.85 (3.21) | -68.42 (2.21) | 5.81 (3.51) | -64.01 (2.24) | 1.35 (2.01) | -65.34 (1.42) | 9.10 (3.84) | -63.25 (2.59) | 0.12 (2.36) | -62.51 (1.80) |
| 95% CI | -12.00, 8.00 | -70.02, -55.73 | -0.50, 12.20 | ‑72.79, -64.05 | -1.15, 12.77 | ‑68.45, ‑59.57 | -2.61, 5.31 | ‑68.14, ‑62.53 | 1.51, 16.69 | ‑68.36, ‑58.15 | -4.53, 4.77 | ‑66.05, ‑58.98 |
| Treatment difference |  |  |  |  |  |  |  |  |  |  |  |  |
| Estimate (SE) |  | -60.87 (6.14) |  | -74.27 (3.87) |  | -69.82 (4.16) |  | -66.68 (2.44) |  | -72.35 (4.63) |  | -62.63 (2.88) |
| 95% CI |  | ‑73.16, -48.58 |  | -81.93, -66.61 |  | ‑78.07, ‑61.57 |  | ‑71.50, ‑61.87 |  | ‑81.50, ‑63.20 |  | ‑68.30, ‑56.97 |
| *P*-value |  | <0.0001 |  | <0.0001 |  | <0.0001 |  | <0.0001 |  | <0.0001 |  | <0.0001 |
| **Non-HDL-C** |  |  |  |  |  |  |  |  |  |  |  |  |
| n | 21 | 41 | 42 | 89 | 32 | 79 | 89 | 180 | 56 | 125 | 111 | 200 |
| LS mean estimate (SE) | -6.87 (4.45) | -53.76 (3.18) | 6.06 (2.93) | -59.74 (2.02) | 4.87 (3.84) | -54.32 (2.44) | -3.48 (1.73) | -56.91 (1.22) | 8.74 (3.27) | -55.14 (2.20) | 1.80 (2.13) | -53.42 (1.62) |
| 95% CI | -15.78, 2.04 | -60.13, -47.40 | 0.26, 11.86 | -63.73, -55.75 | ‑2.75, 12.49 | ‑59.17, ‑49.48 | ‑6.89, ‑0.07 | ‑59.32, ‑54.50 | 2.29, 15.20 | ‑59.48, ‑50.80 | -2.39, 5.98 | ‑56.61, ‑50.24 |
| Treatment difference |  |  |  |  |  |  |  |  |  |  |  |  |
| Estimate (SE) |  | -46.90 (5.47) |  | -65.80 (3.53) |  | -59.19 (4.55) |  | -53.43 (2.10) |  | -63.88 (3.94) |  | -55.22 (2.59) |
| 95% CI |  | -57.85, -35.95 |  | -72.79, -58.80 |  | -68.22, -50.16 |  | -57.57, -49.29 |  | -71.66, -56.10 |  | -60.32, -50.12 |
| *P*-value |  | <0.0001 |  | <0.0001 |  | <0.0001 |  | <0.0001 |  | <0.0001 |  | <0.0001 |
| **ApoB^a^** |  |  |  |  |  |  |  |  |  |  |  |  |
| n | 18 | 39 | 39 | 86 | 31 | 71 | 83 | 165 | 52 | 113 | 106 | 186 |
| LS mean estimate (SE) | -5.60 (4.87) | -50.22 (3.31) | 10.91 (2.92) | -45.64 (1.98) | 7.71 (3.69) | -50.06 (2.44) | -1.87 (2.03) | -43.36 (1.43) | 8.57 (3.46) | -49.23 (2.35) | 1.03 (1.99) | -42.62 (1.52) |
| 95% CI | -15.37, 4.16 | -56.86, -43.59 | 5.12, 16.70 | ‑49.56, -41.71 | 0.39, 15.02 | ‑54.90, -45.23 | ‑5.86, 2.13 | ‑46.19, -40.54 | 1.74, 15.40 | ‑53.87, -44.60 | ‑2.87, 4.94 | ‑45.62, -39.62 |
| Treatment difference |  |  |  |  |  |  |  |  |  |  |  |  |
| Estimate (SE) |  | -44.62 (5.89) |  | -56.55 (3.50) |  | -57.77 (4.42) |  | -41.49 (2.46) |  | -57.81 (4.18) |  | -43.66 (2.44) |
| 95% CI |  | -56.42, -32.81 |  | -63.48, -49.61 |  | -66.54, -49.00 |  | -46.34, -36.65 |  | -66.06, -49.55 |  | -48.45, -38.86 |
| *P*-value |  | <0.0001 |  | <0.0001 |  | <0.0001 |  | <0.0001 |  | <0.0001 |  | <0.0001 |
| **Lp(a)** |  |  |  |  |  |  |  |  |  |  |  |  |
| n | 20 | 41 | 41 | 90 | 32 | 79 | 88 | 177 | 56 | 125 | 111 | 201 |
| Median | 16.36 | -37.50 | 0.00 | -37.50 | 7.57 | -41.67 | 0.27 | -41.94 | -6.17 | -39.17 | 0.00 | -35.00 |
| Q1, Q3 | -2.50,  57.86 | -54.55,  -17.11 | -12.50, 16.67 | -60.53,  -17.47 | -17.22,  19.54 | -59.72,  -24.75 | -13.94, 18.36 | -57.30,  -23.44 | -17.86,  8.99 | -57.81,  -22.22 | -14.58,  16.67 | -54.11,  -16.67 |
| Treatment difference |  |  |  |  |  |  |  |  |  |  |  |  |
| Median difference |  | -53.86 |  | -37.50 |  | -49.24 |  | -42.21 |  | -32.99 |  | -35.00 |
| 95% CI |  | -84.72, -23.01 |  | -49.83, -25.17 |  | -58.95, -39.53 |  | -51.48, -32.93 |  | -42.12, -23.86 |  | -40.60, -29.40 |
| *P*-value |  | <0.0001 |  | <0.0001 |  | <0.0001 |  | <0.0001 |  | <0.0001 |  | <0.0001 |
| **HDL-C** |  |  |  |  |  |  |  |  |  |  |  |  |
| n | 21 | 41 | 42 | 89 | 32 | 79 | 89 | 180 | 56 | 125 | 111 | 200 |
| LS mean estimate (SE) | -4.44 (2.65) | 2.35 (1.89) | -4.80 (1.86) | 6.34 (1.26) | 0.60 (3.58) | 15.35 (2.29) | 5.96 (1.71) | 14.04 (1.21) | 0.60 (1.63) | 4.72 (1.10) | -3.80 (1.18) | 3.81 (0.90) |
| 95% CI | -9.74, 0.86 | -1.43, 6.13 | -8.47,  -1.12 | 3.84, 8.84 | -6.48, 7.69 | 10.82, 19.87 | 2.60, 9.32 | 11.66, 16.41 | -2.61, 3.81 | 2.56, 6.88 | -6.13, -1.47 | 2.03, 5.58 |
| Treatment difference |  |  |  |  |  |  |  |  |  |  |  |  |
| Estimate (SE) |  | 6.79 (3.25) |  | 11.14 (2.23) |  | 14.74 (4.24) |  | 8.08 (2.07) |  | 4.12 (1.96) |  | 7.60 (1.44) |
| 95% CI |  | 0.28, 13.29 |  | 6.72, 15.55 |  | 6.33, 23.15 |  | 4.00, 12.16 |  | 0.25, 7.99 |  | 4.76, 10.44 |
| *P*-value |  | 0.041 |  | <0.0001 |  | 0.0007 |  | 0.0001 |  | 0.037 |  | <0.0001 |

**Supplemental Table 2B:**

| **Lipid parameters, percent change from baseline values** | **High TGs and normal HDL-C** | | | | **High TGs and low HDL-C** | | | | **Normal TGs and normal HDL-C** | | | |
| --- | --- | --- | --- | --- | --- | --- | --- | --- | --- | --- | --- | --- |
|  | **Placebo (N=64)** | | **Evolocumab (N=132)** | | **Placebo (N=125)** | | **Evolocumab (N=264)** | | **Placebo (N=178)** | | **Evolocumab (N=341)** | |
| **VLDL-C** |  |  |  |  |  |  |  |  |  |  |  |  |
| n | 61 | | 130 | | 114 | | 250 | | 167 | | 325 | |
| Median | -10.53 | | -26.67 | | -6.63 | | -26.19 | | 8.33 | | -6.67 | |
| Q1, Q3 | -26.55, 10.29 | | -39.73, -5.10 | | -23.94, 8.74 | | -40.00, -11.48 | | -7.14, 24.49 | | -23.81, 12.50 | |
| Treatment difference |  |  |  |  |  |  |  |  |  |  |  |  |
| Median difference |  |  | -16.14 | |  |  | -19.56 | |  |  | -15.00 | |
| 95% CI |  |  | -25.33, -6.95 | |  |  | -25.79, -13.33 | |  |  | -21.26, -8.74 | |
| *P*-value |  |  | 0.0002 | |  |  | <0.0001 | |  |  | <0.0001 | |
| **Remnant cholesterol** |  |  |  |  |  |  |  |  |  |  |  |  |
| n | 63 | | 130 | | 121 | | 259 | | 167 | | 325 | |
| Median | -9.01 | | -37.90 | | -6.12 | | -40.32 | | 8.33 | | -17.65 | |
| Q1, Q3 | -26.55, 14.29 | | -50.00, -20.88 | | -26.09, 11.61 | | -53.85, -23.91 | | -7.14, 24.49 | | -34.62, 5.88 | |
| Treatment difference |  |  |  |  |  |  |  |  |  |  |  |  |
| Median difference |  |  | -28.89 | |  |  | -34.20 | |  |  | -25.98 | |
| 95% CI |  |  | -39.47, -18.31 | |  |  | -41.52, -26.88 | |  |  | -32.12, -19.84 | |
| *P*-value |  |  | <0.0001 | |  |  | <0.0001 | |  |  | <0.0001 | |
| **TGs** |  |  |  |  |  |  |  |  |  |  |  |  |
| n | 63 | | 130 | | 121 | | 259 | | 167 | | 325 | |
| Median | -8.61 | | -26.10 | | -6.71 | | -26.32 | | 8.70 | | -6.87 | |
| Q1, Q3 | -25.75, 15.83 | | -39.39, -6.28 | | -24.12, 11.40 | | -40.84, -8.72 | | -6.88, 25.25 | | -23.46, 13.74 | |
| Treatment difference |  |  |  |  |  |  |  |  |  |  |  |  |
| Median difference |  |  | -17.49 | |  |  | -19.61 | |  |  | -15.57 | |
| 95% CI |  |  | -26.50, -8.48 | |  |  | -25.99, -13.23 | |  |  | -21.67, -9.47 | |
| *P*-value |  |  | <0.0001 | |  |  | <0.0001 | |  |  | <0.0001 | |

ApoB = apolipoprotein B; CI = confidence interval; HDL-C = high-density lipoprotein cholesterol; IU = international unit; LDL-C = low-density lipoprotein cholesterol; Lp(a) = lipoprotein(a); LS = least squares; non-HDL-C = non-high-density lipoprotein cholesterol; Q2W = every 2 weeks; QM = monthly; SE = standard error; TGs = triglycerides; VLDL‑C = very low-density lipoprotein cholesterol.

^a^ApoB collected only at week 12.

LS means, associated 95% CI and p-values are from a repeated measures linear effects model which includes study, treatment group, scheduled visit, and the interaction of treatment with scheduled visit as covariates. Median difference and 95% CI are obtained from McKean-Schrader algorithm. P-value is obtained from Quade test adjusting for baseline value. Treatment differences use placebo as the reference within each dose frequency. When the calculated LDL-C was <1.0 mmol/L or TGs were >4.5 mmol/L, calculated LDL-C was replaced with ultracentrifugation to inform LDL-C and VLDL-C from the same blood sample, if available.

**Supplemental Table 3. Change from baseline lipid values with evolocumab vs placebo treatment (mean of weeks 10 and 12)**

**Supplemental Table 3A:**

| **Lipid parameters, change from baseline values** | **High TGs and normal HDL-C** | | | | **High TGs and low HDL-C** | | | | **Normal TGs and normal HDL-C** | | | |
| --- | --- | --- | --- | --- | --- | --- | --- | --- | --- | --- | --- | --- |
|  | **Q2W** | | **QM** | | **Q2W** | | **QM** | | **Q2W** | | **QM** | |
|  | **Placebo** | **Evolocumab** | **Placebo** | **Evolocumab** | **Placebo** | **Evolocumab** | **Placebo** | **Evolocumab** | **Placebo** | **Evolocumab** | **Placebo** | **Evolocumab** |
|  | **(N=21)** | **(N=42)** | **(N=43)** | **(N=90)** | **(N=33)** | **(N=81)** | **(N=92)** | **(N=183)** | **(N=62)** | **(N=129)** | **(N=116)** | **(N=212)** |
| **LDL-C, mmol/L** |  |  |  |  |  |  |  |  |  |  |  |  |
| n | 21 | 41 | 42 | 89 | 32 | 79 | 89 | 180 | 56 | 125 | 111 | 200 |
| LS mean estimate (SE) | -0.074 (0.202) | -1.632 (0.143) | 0.112 (0.110) | -1.865 (0.076) | 0.108 (0.121) | -1.627 (0.077) | -0.013 (0.071) | -1.675 (0.050) | 0.137 (0.105) | -1.488 (0.070) | -0.085 (0.069) | -1.650 (0.053) |
| 95% CI | -0.478, 0.331 | ‑1.919,  -1.345 | -0.105, 0.329 | -2.015,  -1.715 | ‑0.132,  0.348 | ‑1.780,  ‑1.474 | ‑0.153,  0.126 | ‑1.773,  ‑1.576 | ‑0.070,  0.344 | ‑1.626,  ‑1.349 | -0.221, 0.051 | ‑1.754, ‑1.547 |
| Treatment difference |  |  |  |  |  |  |  |  |  |  |  |  |
| Estimate (SE) |  | -1.558 (0.248) |  | -1.977 (0.133) |  | -1.735 (0.144) |  | -1.661 (0.086) |  | -1.624 (0.126) |  | -1.565 (0.084) |
| 95% CI |  | -2.054,  -1.062 |  | -2.239,  -1.714 |  | -2.019,  ‑1.450 |  | -1.830,  ‑1.492 |  | -1.874,  ‑1.375 |  | -1.731,  ‑1.400 |
| *P*-value |  | <0.0001 |  | <0.0001 |  | <0.0001 |  | <0.0001 |  | <0.0001 |  | <0.0001 |
| **Non-HDL-C, mmol/L** |  |  |  |  |  |  |  |  |  |  |  |  |
| n | 21 | 41 | 42 | 89 | 32 | 79 | 89 | 180 | 56 | 125 | 111 | 200 |
| LS mean estimate (SE) | -0.252 (0.217) | -1.948 (0.154) | 0.226 (0.129) | -2.243 (0.089) | 0.109 (0.164) | -2.032 (0.104) | -0.156 (0.082) | -2.141 (0.058) | 0.186 (0.109) | -1.595 (0.073) | -0.029 (0.073) | -1.697 (0.055) |
| 95% CI | -0.686, 0.182 | -2.256,  -1.639 | -0.029, 0.481 | -2.419,  ‑2.068 | -0.217, 0.434 | -2.239,  ‑1.825 | -0.317, 0.005 | -2.255,  ‑2.027 | -0.030, 0.402 | -1.740,  -1.451 | -0.172, 0.114 | -1.806,  -1.588 |
| Treatment difference |  |  |  |  |  |  |  |  |  |  |  |  |
| Estimate (SE) |  | -1.696 (0.266) |  | -2.469 (0.155) |  | -2.140 (0.194) |  | -1.984 (0.099) |  | -1.781 (0.132) |  | -1.668 (0.089) |
| 95% CI |  | -2.228,  -1.163 |  | -2.777,  -2.162 |  | -2.526,  -1.755 |  | -2.180,  -1.789 |  | -2.041,  -1.521 |  | -1.842,  -1.493 |
| *P*-value |  | <0.0001 |  | <0.0001 |  | <0.0001 |  | <0.0001 |  | <0.0001 |  | <0.0001 |
| **ApoB, g/L^a^** |  |  |  |  |  |  |  |  |  |  |  |  |
| n | 18 | 39 | 39 | 86 | 31 | 71 | 83 | 165 | 52 | 113 | 106 | 186 |
| LS mean estimate (SE) | -0.068 (0.059) | -0.484 (0.040) | 0.103 (0.031) | -0.449 (0.021) | 0.053 (0.041) | -0.509 (0.027) | -0.034 (0.023) | -0.430 (0.017) | 0.050 (0.030) | -0.396 (0.020) | -0.010 (0.018) | -0.365 (0.014) |
| 95% CI | -0.187, 0.050 | -0.564,  -0.404 | 0.042, 0.165 | -0.491,  -0.407 | -0.029, 0.135 | -0.563,  -0.455 | -0.080, 0.012 | -0.462,  -0.397 | -0.008, 0.108 | -0.436,  -0.357 | -0.046, 0.026 | -0.393,  -0.338 |
| Treatment difference |  |  |  |  |  |  |  |  |  |  |  |  |
| Estimate (SE) |  | -0.415 (0.071) |  | -0.552 (0.037) |  | -0.562 (0.050) |  | -0.395 (0.028) |  | -0.446 (0.036) |  | -0.355 (0.022) |
| 95% CI |  | -0.558,  -0.273 |  | -0.626,  -0.478 |  | -0.661,  -0.464 |  | -0.451,  -0.340 |  | -0.517,  -0.376 |  | -0.399,  -0.311 |
| *P*-value |  | <0.0001 |  | <0.0001 |  | <0.0001 |  | <0.0001 |  | <0.0001 |  | <0.0001 |
| **Lp(a), nmol/L** |  |  |  |  |  |  |  |  |  |  |  |  |
| n | 20 | 41 | 41 | 90 | 32 | 79 | 88 | 177 | 56 | 125 | 111 | 201 |
| Median | 4.0 | -10.5 | 0.0 | -11.5 | 2.0 | -9.0 | 0.3 | -9.5 | -2.0 | -10.5 | 0.0 | -15.0 |
| Q1, Q3 | -0.5, 18.5 | -49.5, -1.0 | -2.5, 3.0 | -31.5, -4.0 | -2.0, 7.5 | -21.0, -3.0 | -3.8, 8.0 | -27.5, -2.5 | -6.8, 2.3 | -25.0, -4.0 | -7.5, 7.0 | -26.5, -3.5 |
| Treatment difference |  |  |  |  |  |  |  |  |  |  |  |  |
| Median difference |  | -14.5 |  | -11.5 |  | -11.0 |  | -9.8 |  | -8.5 |  | -15.0 |
| 95% CI |  | -27.6, -1.4 |  | -18.4, -4.6 |  | -16.8, -5.2 |  | -13.6, -5.9 |  | -11.8, -5.2 |  | -18.5, -11.5 |
| *P*-value |  | <0.0001 |  | <0.0001 |  | <0.0001 |  | <0.0001 |  | <0.0001 |  | <0.0001 |
| **HDL-C, mmol/L** |  |  |  |  |  |  |  |  |  |  |  |  |
| n | 21 | 41 | 42 | 89 | 32 | 79 | 89 | 180 | 56 | 125 | 111 | 200 |
| LS mean estimate (SE) | -0.069 (0.038) | 0.037 (0.027) | -0.063 (0.025) | 0.082 (0.017) | 0.004 (0.030) | 0.134 (0.019) | 0.055 (0.015) | 0.124 (0.010) | 0.006 (0.024) | 0.059 (0.016) | -0.059 (0.017) | 0.048 (0.013) |
| 95% CI | -0.144, 0.006 | -0.017, 0.090 | -0.113,  -0.013 | 0.047, 0.116 | -0.055, 0.064 | 0.096, 0.172 | 0.026, 0.084 | 0.103, 0.145 | -0.041, 0.053 | 0.028, 0.091 | -0.093,  -0.026 | 0.023, 0.074 |
| Treatment difference |  |  |  |  |  |  |  |  |  |  |  |  |
| Estimate (SE) |  | 0.106 (0.046) |  | 0.144 (0.031) |  | 0.130 (0.036) |  | 0.069 (0.018) |  | 0.053 (0.029) |  | 0.108 (0.021) |
| 95% CI |  | 0.014, 0.198 |  | 0.084, 0.205 |  | 0.060, 0.201 |  | 0.033, 0.104 |  | -0.003, 0.110 |  | 0.067, 0.149 |
| *P*-value |  | 0.025 |  | <0.0001 |  | 0.0004 |  | 0.0002 |  | 0.062 |  | <0.0001 |

**Supplemental Table 3B:**

| **Lipid parameters, change from baseline values** | **High TGs and normal HDL-C** | | | | **High TGs and low HDL-C** | | | | **Normal TGs and normal HDL-C** | | | |
| --- | --- | --- | --- | --- | --- | --- | --- | --- | --- | --- | --- | --- |
|  | **Placebo**  **(N=64)** | | **Evolocumab**  **(N=132)** | | **Placebo**  **(N=125)** | | **Evolocumab**  **(N=264)** | | **Placebo**  **(N=178)** | | **Evolocumab**  **(N=341)** | |
| **VLDL-C, mmol/L** |  |  |  |  |  |  |  |  |  |  |  |  |
| n | 61 | | 130 | | 114 | | 250 | | 167 | | 325 | |
| Median | -0.095 | | -0.250 | | -0.073 | | -0.278 | | 0.035 | | -0.030 | |
| Q1, Q3 | -0.270, 0.120 | | -0.415, -0.060 | | -0.250, 0.105 | | -0.465, -0.120 | | -0.045, 0.125 | | -0.130, 0.055 | |
| Treatment difference |  |  |  |  |  |  |  |  |  |  |  |  |
| Median difference |  |  | -0.155 | |  |  | -0.205 | |  |  | -0.065 | |
| 95% CI |  |  | -0.251, -0.059 | |  |  | -0.271, -0.139 | |  |  | -0.095, -0.035 | |
| *P*-value |  |  | 0.0002 | |  |  | <0.0001 | |  |  | <0.0001 | |
| **Remnant cholesterol, mmol/L** |  |  |  |  |  |  |  |  |  |  |  |  |
| n | 63 | | 130 | | 121 | | 259 | | 167 | | 325 | |
| Median | -0.095 | | -0.353 | | -0.070 | | -0.440 | | 0.035 | | -0.085 | |
| Q1, Q3 | -0.270, 0.150 | | -0.470, -0.200 | | -0.255, 0.135 | | -0.655, 0.255 | | -0.040, 0.120 | | -0.185, 0.025 | |
| Treatment difference |  |  |  |  |  |  |  |  |  |  |  |  |
| Median difference |  |  | -0.258 | |  |  | -0.370 | |  |  | -0.120 | |
| 95% CI |  |  | -0.359, -0.156 | |  |  | -0.444, -0.296 | |  |  | -0.152, -0.088 | |
| *P*-value |  |  | <0.0001 | |  |  | <0.0001 | |  |  | <0.0001 | |
| **TGs, mmol/L** |  |  |  |  |  |  |  |  |  |  |  |  |
| n | 63 | | 130 | | 121 | | 259 | | 167 | | 325 | |
| Median | -0.180 | | -0.535 | | -0.170 | | -0.610 | | 0.090 | | -0.070 | |
| Q1, Q3 | -0.615, 0.345 | | -0.900, -0.120 | | -0.590, 0.260 | | -1.025, -0.220 | | -0.080, 0.260 | | -0.260, 0.120 | |
| Treatment difference |  |  |  |  |  |  |  |  |  |  |  |  |
| Median difference |  |  | -0.355 | |  |  | -0.440 | |  |  | -0.160 | |
| 95% CI |  |  | -0.574, -0.136 | |  |  | -0.583, -0.297 | |  |  | -0.230, -0.090 | |
| *P*-value |  |  | <0.0001 | |  |  | <0.0001 | |  |  | <0.0001 | |

ApoB = apolipoprotein B; CI = confidence interval; HDL-C = high-density lipoprotein cholesterol; LDL-C = low-density lipoprotein cholesterol; Lp(a) = lipoprotein(a); LS = least squares; non-HDL-C = non-high-density lipoprotein cholesterol; Q2W = every 2 weeks; QM = monthly; SE = standard error; TGs = triglycerides; VLDL‑C = very low-density lipoprotein cholesterol.

LS means, associated 95% CI and p-values are from a repeated measures linear effects model which includes study, treatment group, scheduled visit, and the interaction of treatment with scheduled visit as covariates. Median difference and 95% CI are obtained from McKean-Schrader algorithm. P-value is obtained from Quade test adjusting for baseline value

^a^ApoB collected only at week 12. When the calculated LDL-C was <1.0 mmol/L or TGs were >4.5 mmol/L, calculated LDL-C was replaced with ultracentrifugation to inform LDL-C and VLDL-C from the same blood sample, if available.

**Supplemental Table 4. Percentage of patients who met each lipid goal with evolocumab vs placebo treatment (mean of weeks 10 and 12)**

| **Attainment of lipid goals** | **High TGs and normal HDL-C** | | | | **High TGs and low HDL-C** | | | | **Normal TGs and normal HDL-C** | | | |
| --- | --- | --- | --- | --- | --- | --- | --- | --- | --- | --- | --- | --- |
|  | **Q2W** | | **QM** | | **Q2W** | | **QM** | | **Q2W** | | **QM** | |
|  | **Placebo** | **Evolocumab** | **Placebo** | **Evolocumab** | **Placebo** | **Evolocumab** | **Placebo** | **Evolocumab** | **Placebo** | **Evolocumab** | **Placebo** | **Evolocumab** |
|  | **(N=21)** | **(N=42)** | **(N=43)** | **(N=90)** | **(N=33)** | **(N=81)** | **(N=92)** | **(N=183)** | **(N=62)** | **(N=129)** | **(N=116)** | **(N=212)** |
| **LDL-C ≥50% reduction from BL and LDL-C <1.4 mmol/L** |  |  |  |  |  |  |  |  |  |  |  |  |
| n | 21 | 41 | 42 | 89 | 32 | 79 | 89 | 180 | 56 | 125 | 111 | 200 |
| Responders, n (%) | 0 (0.0) | 31 (75.6) | 0 (0.0) | 71 (79.8) | 0 (0.0) | 59 (74.7) | 0 (0.0) | 140 (77.8) | 0 (0.0) | 94 (75.2) | 1 (0.9) | 152 (76.0) |
| 95% CI | 0.0, 15.5 | 60.7, 86.2 | 0.0, 8.4 | 70.3, 86.8 | 0.0, 10.7 | 64.1, 83.0 | 0.0, 4.1 | 71.2, 83.2 | 0.0, 6.4 | 67.0, 81.9 | 0.2, 4.9 | 69.6, 81.4 |
| Treatment difference |  |  |  |  |  |  |  |  |  |  |  |  |
| Estimate |  | 75.6 |  | 79.8 |  | 74.7 |  | 77.8 |  | 75.2 |  | 75.1 |
| 95% CI |  | 54.1, 86.2 |  | 67.1, 86.8 |  | 59.6, 83.0 |  | 70.0, 83.2 |  | 64.7, 81.9 |  | 67.6, 80.5 |
| *P*-value |  | <0.0001 |  | <0.0001 |  | <0.0001 |  | <0.0001 |  | <0.0001 |  | <0.0001 |
| **LDL-C ≥50% reduction from BL** |  |  |  |  |  |  |  |  |  |  |  |  |
| n | 21 | 41 | 42 | 89 | 32 | 79 | 89 | 180 | 56 | 125 | 111 | 200 |
| Responders, n (%) | 1 (4.8) | 33 (80.5) | 0 (0.0) | 78 (87.6) | 0 (0.0) | 63 (79.7) | 0 (0.0) | 148 (82.2) | 1 (1.8) | 97 (77.6) | 2 (1.8) | 166 (83.0) |
| 95% CI | 0.8, 22.7 | 66.0, 89.8 | 0.0, 8.4 | 79.2, 93.0 | 0.0, 10.7 | 69.6, 87.1 | 0.0, 4.1 | 76.0, 87.1 | 0.3, 9.4 | 69.5, 84.0 | 0.5, 6.3 | 77.2, 87.6 |
| Treatment difference |  |  |  |  |  |  |  |  |  |  |  |  |
| Estimate |  | 75.7 |  | 87.6 |  | 79.7 |  | 82.2 |  | 75.8 |  | 81.2 |
| 95% CI |  | 52.7, 85.8 |  | 75.8, 93.0 |  | 65.0, 87.1 |  | 74.7, 87.1 |  | 64.7, 82.4 |  | 73.8, 86.0 |
| *P*-value |  | <0.0001 |  | <0.0001 |  | <0.0001 |  | <0.0001 |  | <0.0001 |  | <0.0001 |
| **LDL-C <1.4 mmol/L** |  |  |  |  |  |  |  |  |  |  |  |  |
| n | 21 | 41 | 42 | 89 | 32 | 79 | 89 | 180 | 56 | 125 | 111 | 200 |
| Responders, n (%) | 2 (9.5) | 32 (78.0) | 0 (0.0) | 74 (83.1) | 0 (0.0) | 67 (84.8) | 4 (4.5) | 151 (83.9) | 3 (5.4) | 104 (83.2) | 6 (5.4) | 163 (81.5) |
| 95% CI | 2.7, 28.9 | 63.3, 88.0 | 0.0, 8.4 | 74.0, 89.5 | 0.0, 10.7 | 75.3, 91.1 | 1.8, 11.0 | 77.8, 88.5 | 1.8, 14.6 | 75.7, 88.7 | 2.5, 11.3 | 75.5, 86.3 |
| Treatment difference |  |  |  |  |  |  |  |  |  |  |  |  |
| Estimate |  | 68.5 |  | 83.1 |  | 84.8 |  | 79.4 |  | 77.8 |  | 76.1 |
| 95% CI |  | 44.2, 80.6 |  | 70.8, 89.5 |  | 70.5, 91.1 |  | 70.5, 84.8 |  | 65.9, 84.4 |  | 67.7, 81.7 |
| *P*-value |  | <0.0001 |  | <0.0001 |  | <0.0001 |  | <0.0001 |  | <0.0001 |  | <0.0001 |
| **LDL-C <1.0 mmol/L** |  |  |  |  |  |  |  |  |  |  |  |  |
| n | 21 | 41 | 42 | 89 | 32 | 79 | 89 | 180 | 56 | 125 | 111 | 200 |
| Responders, n (%) | 0 (0.0) | 26 (63.4) | 0 (0.0) | 58 (65.2) | 0 (0.0) | 47 (59.5) | 0 (0.0) | 117 (65.0) | 0 (0.0) | 88 (70.4) | 1 (0.9) | 120 (60.0) |
| 95% CI | 0.0, 15.5 | 48.1, 76.4 | 0.0, 8.4 | 54.8, 74.3 | 0.0, 10.7 | 48.5, 69.6 | 0.0, 4.1 | 57.8, 71.6 | 0.0, 6.4 | 61.9, 77.7 | 0.2, 4.9 | 53.1, 66.5 |
| Treatment difference |  |  |  |  |  |  |  |  |  |  |  |  |
| Estimate |  | 63.4 |  | 65.2 |  | 59.5 |  | 65.0 |  | 70.4 |  | 59.1 |
| 95% CI |  | 41.7, 76.4 |  | 51.9, 74.3 |  | 44.1, 69.6 |  | 56.7, 71.6 |  | 59.7, 77.7 |  | 51.1, 65.7 |
| *P*-value |  | <0.0001 |  | <0.0001 |  | <0.0001 |  | <0.0001 |  | <0.0001 |  | <0.0001 |
| **Non-HDL-C <2.6 mmol/L** |  |  |  |  |  |  |  |  |  |  |  |  |
| n | 21 | 41 | 42 | 89 | 32 | 79 | 89 | 180 | 56 | 125 | 111 | 200 |
| Responders, n (%) | 6 (28.6) | 37 (90.2) | 2 (4.8) | 81 (91.0) | 5 (15.6) | 68 (86.1) | 5 (5.6) | 157 (87.2) | 20 (35.7) | 114 (91.2) | 36 (32.4) | 185 (92.5) |
| 95% CI | 13.8, 50.0 | 77.5, 96.1 | 1.3, 15.8 | 83.3, 95.4 | 6.9, 31.8 | 76.8, 92.0 | 2.4, 12.5 | 81.6, 91.3 | 24.5, 48.8 | 84.9, 95.0 | 24.4, 41.6 | 88.0, 95.4 |
| Treatment difference |  |  |  |  |  |  |  |  |  |  |  |  |
| Estimate |  | 61.7 |  | 86.2 |  | 70.5 |  | 81.6 |  | 55.5 |  | 60.1 |
| 95% CI |  | 36.8, 77.6 |  | 72.8, 91.8 |  | 51.8, 81.1 |  | 72.7, 86.8 |  | 41.0, 67.4 |  | 49.9, 68.6 |
| *P*-value |  | <0.0001 |  | <0.0001 |  | <0.0001 |  | <0.0001 |  | <0.0001 |  | <0.0001 |
| **Non-HDL-C <2.2 mmol/L** |  |  |  |  |  |  |  |  |  |  |  |  |
| n | 21 | 41 | 42 | 89 | 32 | 79 | 89 | 180 | 56 | 125 | 111 | 200 |
| Responders, n (%) | 2 (9.5) | 31 (75.6) | 0 (0.0) | 74 (83.1) | 1 (3.1) | 64 (81.0) | 3 (3.4) | 138 (76.7) | 10 (17.9) | 111 (88.8) | 12 (10.8) | 170 (85.0) |
| 95% CI | 2.7, 28.9 | 60.7, 86.2 | 0.0, 8.4 | 74.0, 89.5 | 0.6, 15.7 | 71.0, 88.1 | 1.2, 9.4 | 70.0, 82.2 | 10.0, 29.8 | 82.1, 93.2 | 6.3, 18.0 | 79.4, 89.3 |
| Treatment difference |  |  |  |  |  |  |  |  |  |  |  |  |
| Estimate |  | 66.1 |  | 83.1 |  | 77.9 |  | 73.3 |  | 70.9 |  | 74.2 |
| 95% CI |  | 41.6, 78.7 |  | 70.8, 89.5 |  | 61.8, 85.5 |  | 64.3, 79.3 |  | 57.2, 80.0 |  | 65.1, 80.4 |
| *P*-value |  | <0.0001 |  | <0.0001 |  | <0.0001 |  | <0.0001 |  | <0.0001 |  | <0.0001 |
| **ApoB <80 mg/dL^a^** |  |  |  |  |  |  |  |  |  |  |  |  |
| n | 19 | 39 | 39 | 86 | 32 | 71 | 87 | 167 | 52 | 114 | 106 | 186 |
| Responders, n (%) | 10 (52.6) | 35 (89.7) | 4 (10.3) | 75 (87.2) | 7 (21.9) | 63 (88.7) | 22 (25.3) | 142 (85.0) | 28 (53.8) | 107 (93.9) | 51 (48.1) | 173 (93.0) |
| 95% CI | 31.7, 72.7 | 76.4, 95.9 | 4.1, 23.6 | 78.5, 92.7 | 11.0, 38.8 | 79.3, 94.2 | 17.3, 35.3 | 78.8, 89.6 | 40.5, 66.7 | 87.9, 97.0 | 38.8, 57.5 | 88.4, 95.9 |
| Treatment difference |  |  |  |  |  |  |  |  |  |  |  |  |
| Estimate |  | 37.1 |  | 77.0 |  | 66.9 |  | 59.7 |  | 40.0 |  | 44.9 |
| 95% CI |  | 13.0, 58.9 |  | 61.1, 85.2 |  | 47.5, 79.0 |  | 47.9, 68.9 |  | 25.9, 53.7 |  | 34.4, 54.6 |
| *P*-value |  | 0.003 |  | <0.0001 |  | <0.0001 |  | <0.0001 |  | <0.0001 |  | <0.0001 |
| **ApoB <65 mg/dL** |  |  |  |  |  |  |  |  |  |  |  |  |
| n | 19 | 39 | 39 | 86 | 32 | 71 | 87 | 167 | 52 | 114 | 106 | 186 |
| Responders, n (%) | 3 (15.8) | 32 (82.1) | 0 (0.0) | 65 (75.6) | 2 (6.3) | 56 (78.9) | 2 (2.3) | 113 (67.7) | 13 (25.0) | 99 (86.8) | 25 (23.6) | 152 (81.7) |
| 95% CI | 5.5, 37.6 | 67.3, 91.0 | 0.0, 9.0 | 65.5, 83.4 | 1.7, 20.1 | 68.0, 86.8 | 0.6, 8.0 | 60.2, 74.3 | 15.2, 38.2 | 79.4, 91.9 | 16.5, 32.5 | 75.5, 86.6 |
| Treatment difference |  |  |  |  |  |  |  |  |  |  |  |  |
| Estimate |  | 66.3 |  | 75.6 |  | 72.6 |  | 65.4 |  | 61.8 |  | 58.1 |
| 95% CI |  | 40.0, 79.9 |  | 62.1, 83.4 |  | 55.0, 81.7 |  | 56.0, 72.2 |  | 46.7, 72.8 |  | 47.3, 66.7 |
| *P*-value |  | <0.0001 |  | <0.0001 |  | <0.0001 |  | <0.0001 |  | <0.0001 |  | <0.0001 |

ApoB = apolipoprotein B; BL = baseline; CI = confidence interval; HDL-C = high-density lipoprotein cholesterol; LDL-C = low-density lipoprotein cholesterol; non-HDL-C = non-high-density lipoprotein cholesterol; Q2W = every 2 weeks; QM = monthly; TGs = triglycerides.

^a^ApoB collected only at week 12.

Note: The 95% CIs are calculated using the Wilson score method. Treatment differences use placebo as the reference within each dose frequency. *P*-values are based on Cochran-Mantel-Haenszel tests stratified by study. If a value was missing, the patient was considered to have not met the relevant goal. When the calculated LDL-C was <1.0 mmol/L or TGs were >4.5 mmol/L, calculated LDL-C was replaced with ultracentrifugation to inform LDL-C and VLDL-C from the same blood sample, if available.
